# Molecular dynamics simulation of the follicle-stimulating hormone receptor. Understanding the conformational dynamics of receptor variants at positions N680 and D408 from *in silico* analysis

**DOI:** 10.1371/journal.pone.0207526

**Published:** 2018-11-21

**Authors:** Eduardo Jardón-Valadez, Derik Castillo-Guajardo, Iván Martínez-Luis, Rubén Gutiérrez-Sagal, Teresa Zariñán, Alfredo Ulloa-Aguirre

**Affiliations:** 1 Departamento de Recursos de la Tierra, Universidad Autónoma Metropolitana, Unidad Lerma, Estado de México, Mexico; 2 Departamento de Ciencias Ambientales Universidad Autónoma Metropolitana, Unidad Lerma, Estado de México, Mexico; 3 Red de Apoyo a la Investigación, National University of Mexico (UNAM) and Instituto Nacional de Ciencias Médicas y Nutrición SZ, Mexico City, Mexico; Hong Kong University of Science and Technology, HONG KONG

## Abstract

Follicle-stimulating hormone receptor (FSHR) is a G-protein coupled receptor (GPCR) and a prototype of the glycoprotein hormone receptors subfamily of GPCRs. Structural data of the FSHR ectodomain in complex with follicle-stimulating hormone suggests a “pull and lift” activation mechanism that triggers a conformational change on the seven α-helix transmembrane domain (TMD). To analyze the conformational changes of the FSHR TMD resulting from sequence variants associated with reproductive impairment in humans, we set up a computational approach combining helix modeling and molecular simulation methods to generate conformational ensembles of the receptor at room (300 K) and physiological (310 K) temperatures. We examined the receptor dynamics in an explicit membrane environment of polyunsaturated phospholipids and solvent water molecules. The analysis of the conformational dynamics of the functional (N680 and S680) and dysfunctional (mutations at D408) variants of the FSHR allowed us to validate the FSHR-TMD model. Functional variants display a concerted motion of flexible intracellular regions at TMD helices 5 and 6. Disruption of side chain interactions and conformational dynamics were detected upon mutation at D408 when replaced with alanine, arginine, or tyrosine. Dynamical network analysis confirmed that TMD helices 2 and 5 may share communication pathways in the functional FSHR variants, whereas no connectivity was detected in the dysfunctional mutants, indicating that the global dynamics of the FSHR was sensitive to mutations at amino acid residue 408, a key position apparently linked to misfolding and variable cell surface plasma membrane expression of FSHRs with distinct mutations at this position.

## Introduction

Follicle-stimulating hormone (FSH) or follitropin is a glycoprotein hormone synthesized by the anterior pituitary gland. By binding to and activating its cognate receptor, the FSH receptor (FSHR), this hormone plays a key role in the control of gonadal function. The FSHR is a G protein-coupled receptor (GPCR) that belongs to the conserved Class A (Rhodopsin-like) family of the GPCR superfamily [1–3]. As other structurally related glycoprotein hormone receptors, the luteinizing hormone-chorionic gonadotropin receptor (LHCGR) and the thyroid-stimulating hormone receptor (TSHR), the FSHR is composed of a large NH_2_-terminal extracellular domain or ectodomain, where recognition and binding of its cognate ligand occur [2]. The COOH-terminal end of the ectodomain includes the signal specificity subdomain or hinge region, which structurally links the leucine-rich ectodomain with the seven α-helix transmembrane (TM) domain (TMD). The activation mechanism of the FSHR relies on a complex conformational change and interaction of the ectodomain (FSHR_ED_) with the TMD, which occurs *via* the hinge region [4]. The TMD ends with a carboxyl-terminal extension (Ctail) at the cytoplasmic side, which contains several motifs and residues important for receptor trafficking and function [2].

Structural data of FSH in complex with the entire FSHR_ED_ [4], indicates that a sulfated tyrosine residue (sY335) located in the hinge region accommodates into a well suited pocket located in the interface of the α- and β-subunits of the hormone and that is formed after hormone binding to the receptor ectodomain [4, 5]. This movement of the hinge region “unlocks” its inhibitory effect on the TMD, leading to conformational changes of the latter and eventually to receptor activation [4, 5]. Although important advances in the structural elucidation of the FSHR_ED_ and its role on FSHR activation have been achieved during the last 10 years [4–6], our understanding on the mechanisms subserving FSHR activation remain largely unknown due, in part, to the scarce number of known structures of human GPCRs [3, 7–10]. Therefore, computational methods emerge as a complementary strategy to improve our understanding of the structure-function relationships of this type of membrane receptors.

Jiang et al. (2014) proposed a homology model for the seven TM helices of the FSHR using the β2-adrenergic receptor as master template, and the rhodopsin and A2A-adenosine receptor as supplementary templates [5]. The cavity at the top half of the receptor included T449, I411, L415, V450, S453, Y530, L537, S589, M585, H615, I588, S589, K608 and V612, which in principle could work as a binding pocket. Indeed, the structural features of the model were consistent with previews reports. For example, site-directed mutagenesis experiments suggested that T449 was essential for binding an allosteric agonist to the FSHR [11]; residues Y511 and K513, located in the second extracellular loop (EL2), were identified as conserved residues in known GPCR crystal structures [5]. Nonetheless, the homology strategy for modeling GPCRs might underestimate the structural differences among members of a given family, since the coordinates of a template (or multiple templates) are transferred to the model assuming similarity of structures through conserved sequences. To overcome some of the caveats of the homology modeling, the GPCR-I-Tasser [12] was proposed, among other strategies, as a hybrid protocol using *ab-initio* modeling of the TM helices and experimental data. In this approach, instead of assigning coordinates from the template, contact maps and residue orientations suggested by mutagenesis experiments are converted into 3D restraints in order to reduce the degrees of freedom in the GPCR structure computer simulation [12]. Thus, the modeling procedure of the seven TM helices of the human FSHR using the hybrid approach may provide a starting structure to study conformational changes and to analyze more deeply the impact of point mutations in the TMDs.

Studies on membrane protein receptors using computational approaches include molecular dynamics (MD) methods [13]. The main result of a MD calculation is a simulation trajectory, which contains the coordinates of the system particles as a function of time. By analyzing atom positions, it is possible to calculate averages over time for thermodynamic, structural or dynamical properties [14, 15]. Moreover, the simulation results can be compared against experimental data since, in the heart of the algorithms to solve the motion equations, the potential energy function is built from atomistic properties as well as macroscopic properties measured experimentally [15]. Molecular dynamics studies on membrane proteins have provided meaningful insights on protein function, binding sites interactions, receptor activation, and protein folding, among other important processes, from a biological perspective. In addition, MD methods have been used as a procedure to refine GPCR models using the all atom force field approximation [16–18]. One advantage of the MD approach is the possibility of analyzing the conformation dynamics considering the molecular environment of the protein, including the lipid bilayer, the water solvent, and ions, concurrently interacting at physiological temperature and normal pressure [19].

Mutations on the human FSHR have allowed identification of amino acid residues important for receptor function. For example, the loss-of-function, naturally occurring mutation D408Y found in patients with hypergonadotropic amenorrhea has been associated with intracellular trapping of the receptor probably due to misfolding and impaired upward traffic from the endoplasmic reticulum to the cell surface plasma membrane [20, 21]. Expression of the most common and best studied functional variants of the wild-type (WT) FSHR resulting from single nucleotide polymorphisms (SNP) with either alanine or threonine at position 307, and asparagine or serine at position 680 (which are expressed in strong linkage disequilibrium) also have been studied [22, 23]. Interestingly, the expression of the A307/S680 FSHR variant has been associated with variations in the sensitivity of the FSHR to its cognate ligand and the target cell response to FSH stimulation *in vitro* [24, 25] and *in vivo* [26, 27] among other biological effects. Thus, it is expected that a FSHR model sensitive to point mutations may help to unveil structural and, more importantly, functional effects imposed by particular amino acid substitutions on residues D408 and/or N680, corresponding to a FSHR dysfunctional mutant and functional variant, respectively.

In the present study, we applied a MD set up to determine the conformational dynamics of the FSHR protein in a lipid bilayer environment (of polyunsaturated fatty acid tails), at room (27°C) and physiological (37°C) temperatures. More specifically, our primary objective was to evaluate the impact of point mutations on the structure and conformational dynamics of the FSHR TMD. To this end, we analyzed the trajectories generated for the WT FSHR (I1 variant) as well as the functional S680 variant (I2 variant), and the loss-of-function D408Y (M3 mutant). The interhelical interactions of the carboxylate group of D408 were further explored by replacing its side chains atoms with alanine or arginine in the D408A (M1 mutant) and D408R (M2 mutant) mutant FSHRs. Although the overall structure was well preserved, the conformational dynamics was distinguishable between the functional FSHR variants and dysfunctional mutants. The protein dynamics was indeed reproducible at room and physiological temperatures, suggesting that our MD protocol may generate consistent results for a complementary model validation of a membrane protein receptor. The refinement MD procedure and analysis described herein, may be useful to analyze the conformational dynamics of other members of the glycoprotein hormone receptors family, such as the LHCGR and the TSHR.

## Methods

### *In vitro* studies

#### Construction of S680 and D408A/R/Y FSHRs

**Fig 1:**
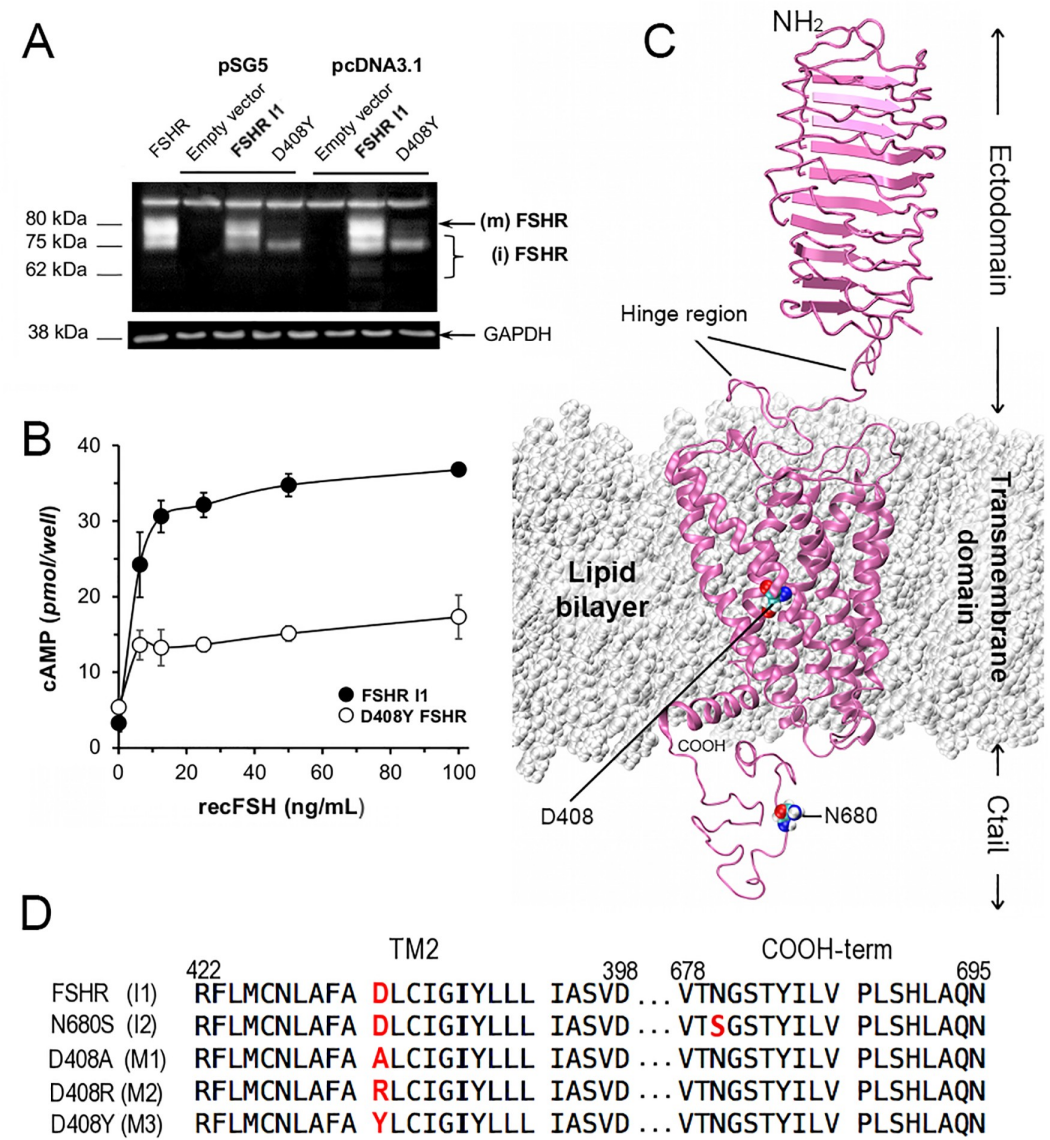
Functional and structural identification of the WT FSHR variant I1, and M3 mutant (D408Y). (A) Representative Western blot of FSHR protein variant I1 (WT FSHR) and mutant M3 (D408Y). The immunoblot shows the relevant portion of an autoradiogram in which the mature [80 kDA (m)], plasma membrane-expressed, fully glycosylated form of the D408Y mutant is considerably reduced compared with that of the WT FSHR I1 variant. The blot shows the migration of FSHRs from protein extracts of HEK293 cells transiently transfected with the WT or mutant FSHR cDNAs inserted either in the pSG5 or pcDNA3.1 vectors. The first lane from left to right, shows the migration of the WT FSHR I1 variant from HEK293 cells stably expressing the receptor. The immunoblot also shows that the majority of the D408Y mutant FSHR is present as immature, intracellular forms [kDa ≤ 75; (i)]. (B) FSH-stimulated production of cAMP by cells expressing the WT or D408Y FSHRs. Compared to the WT receptor, cAMP production was significantly reduced in the M3 mutant (areas under the FSH-stimulated dose-response cAMP curve (pmol/well) = 8218 ± 275 and 4224 ± 346 (means ± SEM) for the WT and M3 FSHR, respectively (p<0.01; Student’s *t* test). (C) Structure domains of the FSHR (magenta ribbons) in the context of the membrane lipid bilayer (white spheres). The seven helix transmembrane domain corresponds to the model of the I1 variant in the lipid bilayer, and the ectodomain was taken from the crystal structure PDB:1XDW [6]. Solvent water molecules were not represented for clarity. Also shown are atoms for residues D408 and N680 depicted as spheres and colored by atom names using the code: C-cyan, O-red, N-blue, and H-white. The hinge region links the transmembrane domain and the ectodomain. (D) Sequence alignments showing the location of the point mutations at the TM helix 2 and the Ctail of the FSHR variants I1 and I2, and mutants M1-3.

Construction of the FSHR variant and mutants was performed employing the full-length WT human FSHR cDNA [28] [GenBank Accession Number S59900] cloned into the mammalian expression vector pSG-5 or pcDNA3.1 (Invitrogen, Waltham, MA, USA). Residues at positions 680 (in the Ctail) or 408 (in TMD helix 2) (Fig 1C) were individually replaced with serine (FSHR N680S) or alanine, arginine or tyrosine (FSHR D408A, D408R, D408Y, respectively) by site directed mutagenesis, cloned into the pSG5 and/or pcDNA3.1 vectors and transiently expressed in human embryonic kidney 293 (HEK293) cells by liposome-mediated endocytosis, as previously described [18]. Forward and reverse mutagenic oligonucleotide primers (Eurofins, Ebersberg, Germany) were designed following the cDNA sequence reported for the testicular WT FSHR [28] (Table A in S1 Text). The identity of all cDNA constructs and the correctness of the PCR yielded sequences were verified by DNA sequencing using the 3500xL automated genetic analyzer (Applied Biosystems, Waltham, MA, USA). For transfection, large scale plasmid DNAs were prepared using an Endofree maxiprep kit (Qiagen, Mexico City, Mexico).

#### Measurement of cAMP production

Forty-eight hours after transfection with the WT and D408Y FSHR cDNAs (cloned into the pSG5 plasmid), cells (in 24-well dishes) were washed with DMEM-5% fetal-calf serum and then stimulated with increasing doses of human recombinant FSH (Merck-Serono, Mexico City, Mexico) in the presence of 0.125 mM 3-isobutyl-methyl-xanthine (Sigma Aldrich, Mexico City, Mexico). At the end of the incubation period (2 h), the medium was removed and total (extracellular plus intracellular) cAMP accumulation was measured by radioimmunoassay as reported previously [29].

#### Reporter gene assay

For the reporter gene assay, HEK293 cells were transiently cotransfected with the WT or mutant (D408A or D408R) FSHR cDNAs (cloned into the pSG5 plasmid) and the cAMP-sensitive pSOMLuc reporter plasmid, employing a previously described procedure [30]. After a 6 h incubation period in the presence or abscence of increasing doses of recombinant human FSH, the cells were lysed and the luciferase activity was measured using a luciferase assay system (Promega Corp., Madison, WI, USA). The light produced was measured in a luminescence counter and expressed as fold increase over basal.

#### FSHR immunoblotting

After sodium dodecyl sulfate polyacrylamide gel electrophoresis (SDS-PAGE) (7.5%), Western blotting of whole cell lysates from HEK293 cells expressing the WT or mutant FSHRs was performed employing as primary antibody the highly specific anti-human FSHR antibody mAb106.105 [31] and the secondary anti-mouse IgG horseradish peroxidase conjugate (Biosource International, Armadillo, CA, USA), as previously described [32]. Development of the signal was performed employing the Pierce ECL Western Blotting detection kit (Rockford, IL, USA). A reprobed membrane with a 1:10000 anti-glyceraldehyde-3-phosphate dehidrogenase (GAPDH) antibody (Sigma) and 1:15000 goat-anti-mouse IgG conjugated with horseradish peroxidase (Biosource) was used to confirm equal protein gel loading.

### *In silico* studies

#### Receptor modeling

The initial receptor coordinates were taken from the FSHR model previously reported [18]. Briefly, the modeling procedure was based on the GPCR-I-TASSER platform [12], selecting the highest scored model for subsequent refinement by MD simulation using the all-atom force field approximation [16]. We chose the GPCR-I-TASSER protocol because it was designed to overcome the lack of known protein structures (templates) in homology-based methods of the GPCR superfamily. The modeling protocol includes multiple sequence alignments over a GPCR-specific database containing residue contact maps, distance restraints, and residue orientations as reported through experimental measurements [33]. One additional advantage of the GPCR-I-TASSER relies on the *ab-initio* helix modeling whenever homology templates are unavailable, which improves the TM-score for helical domains of unknown structures of GPCR [12]. Hence, we used for the initial coordinates the model with the highest TM-score (0.4 ± 0.14) according to the results of the GCPR-I-TASSER server for the protein fragment from D317 to N695. The receptor model was inserted in a pre-equilibrated lipid bilayer of 1-stearoyl-2-docosahexaenoyl-sn-glycero-3-phosphocholine (SDPC) molecules obtained from previous simulations of a GPCR crystal structure [19]. Importantly, interhelical water molecules stable in the squid rhodopsin set up [34] were preserved for the present FSHR model since they are associated to a functional role in the GPCR activation process [35–39]. Disulfide bonds were defined between C338-C356 and C442-C517. Palmitoylated tails were included in C644 and C646 by forming tioester bonds [40]. The protein structure was relaxed for approximately 20 ns of MD simulation [18].

#### Preparation of FSHR mutants and variants

We proposed modifications of the interhelical interactions and electrostatics at position D408 by replacing the carboxylate group by a neutral side chain in D408A (M1 mutant), a basic side chain in D408R (M2 mutant), and a polar group in D408Y (M3 mutant). The D408Y mutation was previously described as a naturally occurring mutation causing hypergonadotropic amenorrhea [20]. The D408Y and D408A mutations lead to reduced surface cell plasma membrane expression and impaired signaling due to intracellular trapping of the mutant receptor in an immature form (Fig 1A and 1B and Fig A in S1 Text), whereas the D408R FSHR mutant is subnormally expressed at the plasma membrane but exhibits severely impaired signaling (Fig A. in S1 Text). To assess the impact of loss of function mutations in the interhelical region (Fig 1C), models for two functional variants of the WT FSHR were generated, namely I1 variant with N680 and I2 variant with S680 (Fig 1D) in the intracellular Ctail. The expression and function of these FSHR variants have been previously tested *in vitro* [24, 25]. The main difference between these variants is that the S680 FSHR stimulates more slowly second messenger production [25] and internalizes faster [24] than the N680 variant when exposed to agonist. All FSHR models were prepared and simulated using the same computational protocol.

#### Molecular dynamics

**Fig 2:**
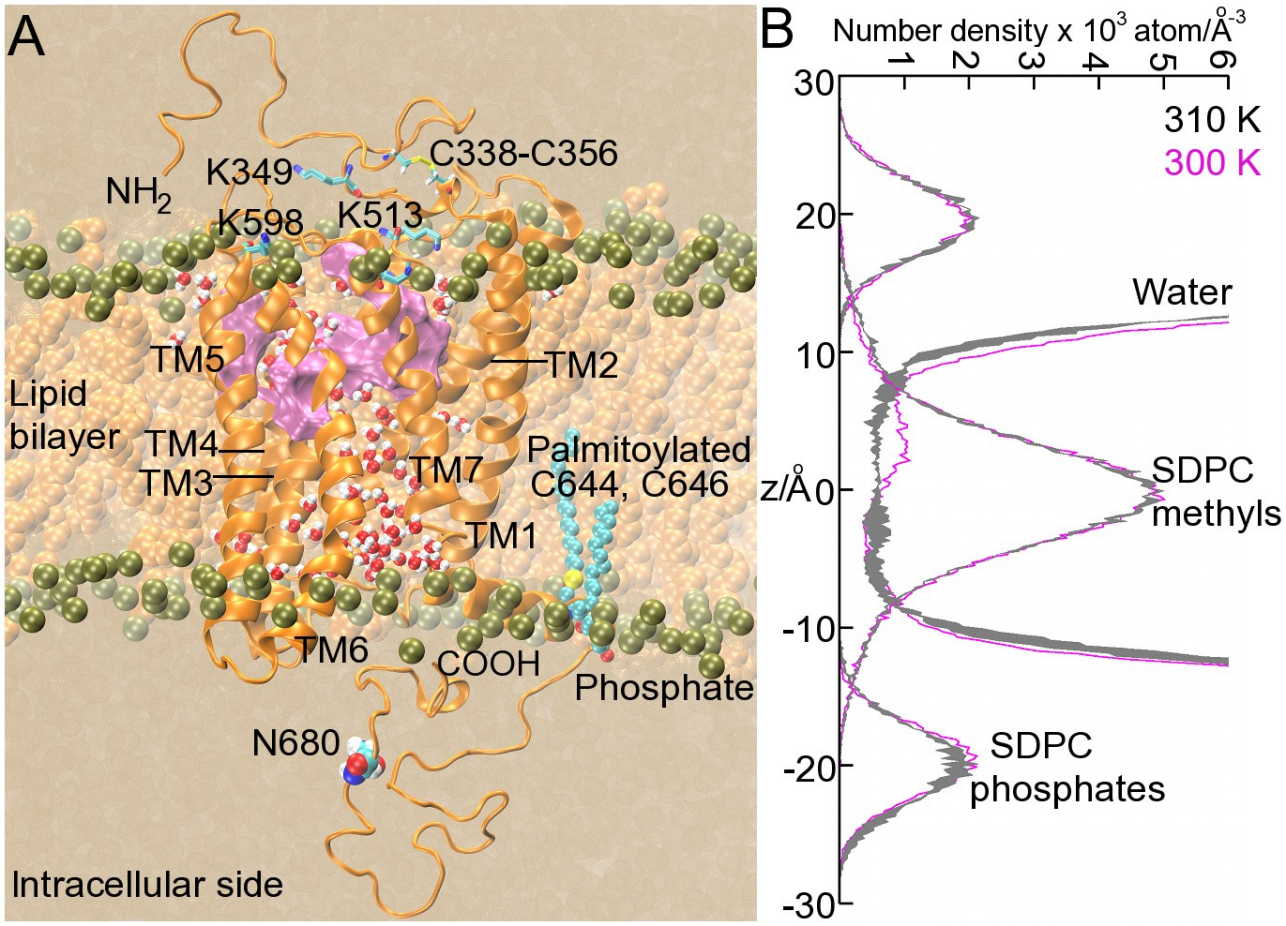
Simulation box including the FSHR and the lipid bilayer. (A). Snapshot at the end of the MD trajectory R0 for the I1 FSHR model. The upper surface in the interhelical region (colored in magenta) corresponds to the putative binding pocket with residues T449, I411, L415, V450, S453, Y530, L537, S589, M585, H615, I588, S589, K608 and V612. The receptor backbone is depicted as orange ribbons; water molecules in the interhelical region are depicted as spheres, with hydrogen in white and oxygen in red; phosphorous atoms are depicted as bronze spheres. In the background, the lipid hydrocarbon tails are depicted as orange spheres. Water is depicted as a continuum solvent in ochre color. (B) Number density across the normal bilayer for water, phosphate and terminal methyl groups, of the lipid heads and tails of the SDPC molecules, respectively. Solid gray lines represent density profiles for the I1 variant at 310 K (line width corresponding to ± standard deviation), and magenta lines at 300 K. The hydrophobic core of the bilayer was defined using the SDPC methyl groups distribution and interfaces using the SDPC phosphate groups distributions. Water molecules in the interhelical region formed a channel communicating the extra- and intracellular sides.

The simulation box included a bilayer of SDPC lipid molecules, water molecules, sodium ions for system neutrality, and the FSHR receptor (Fig 2A). All simulations were performed with the NAMD 2.11 software [41]. To eliminate any repulsive contact, we performed 10 K steps of energy minimization and subsequently 20 ps of simulation at 300 K and constant volume conditions (NVT ensemble). The simulation trajectories were prolonged for ~70 ns for each system at constant pressure conditions (NPT ensemble). A Langevin dynamics to maintain a constant temperature and a Nosé-Hoover Langevin piston to maintain constant pressure of 1 bar were applied [42, 43]; pressure was controlled anisotropically, i.e. cell dimensions were allowed to fluctuate independently in the x-, y-, and z-directions for a proper equilibration of the membrane bilayer [44–46]. Simulation trajectories for each system were generated at temperatures of 310K and 300K for sampling the protein configurational space at physiological temperature and laboratory conditions, respectively. Additional trajectories of ~20 ns were generated at 310 K using as initial conditions time frames (including coordinates and velocities) at ~30 ns and ~50 ns of the main trajectory; these are hereinafter referred to as R0, R1 and R2 runs for the main trajectory, and the replicated trajectories starting at ~30 ns and ~50 ns, respectively. The R1 and R2 trajectories were generated for evaluation of statistical significance of results. Therefore, calculations correspond to averages for three replicas: the last 20 ns of trajectory R0, 20 ns of R1, and 20 ns of R2 (see Table B in S1 Text). The all-atom CHARMM36 force field parameter set was used for the protein [47] and lipids [45]. Water molecules were modeled using the TIP3P model [48]. A multiple time-step scheme for integration of the motion equations was used, 2 fs for the short non-bonding interactions and 4 fs for the electrostatic interactions. The particle-mesh Ewald method was employed to calculate the electrostatic interactions with a tolerance of 10^−6^ for the direct part of the Ewald sum, a fourth-order interpolation scheme, and a grid spacing of ~1.0 Å for each box side [49]. All bond lengths involving hydrogen atoms were constrained using the SHAKE algorithm [50]. Graphics and scripts for analysis were used as implemented in the Visual Molecular Dynamics platform [51], whereas other scripts were developed in house for preparing data files and plots.

#### Trajectory analysis

A set of analysis was performed to evaluate the impact of a point mutation in the TM domains of the FSHR structure, as well as the overall conformational dynamics, in functional and dysfunctional phenotypes. The structure integrity was assessed by analyzing the root mean square deviation (RMSD) of the backbone atoms in the α-helical domains and root mean square fluctuations (RMSF) of the Cα atoms. Contact maps were calculated to identify changes in protein interactions at position 408 of the I1 and I2 variants, and M1-3 mutants. Secondary structure analysis were performed with the DSSP software [52] as implemented in GROMACS 5.1 [53], which identify patters of H-bonds along the protein structure according to a secondary structure dictionary [54]. Density profiles were calculated to determine the bilayer topology and water-lipid interfaces as well as the hydrophobic core of the bilayer, which is the molecular environment stabilizing the receptor model structure and modulating its conformational dynamics.

Variance-covariance matrices were calculated for the Cα atoms of the TM domain, including D358 to C646, after a RMSD fitting of the receptor conformation at time *t* on top of the conformation at time 0, for time frames taken every 4 ps for trajectories R0-2 [55]. Principal component analysis (PCA) was performed in order to summarize the main fluctuations using the first principal component axis [56]. The GROMACS 5.1 analysis packages were used for the PCA calculations [53]. By projecting the MD trajectories over the first principal component (PC1) we could identify the global collective motion along the axis containing the main conformational variability. Then, we parametrized the motion of specific Cα atoms, located at the bilayer interfaces in the upper and lower leaflets of TM helices, and helix 8, by a multiple linear regression technique with categorical (dummy) variables. The parameters that better described the motions of the Cα atoms in trajectories R0-2 were used to detect significant differences in the motion of TM helices among the functional and dysfunctional phenotypes explored (see Table C and Fig L in S1 Text).

Community network analysis was performed for the Cα atoms using the normalized variance-covariance matrix for the full length of trajectories at 300 and 310 K, collecting time frames every 100 ps, in order to identify concerted motions of the TM domains [57]. In the network, clusters of atoms that are highly intra-correlated but loosely inter-correlated are decomposed into communities using the Girvan-Newman algorithm [58]. Communities may be connected in the network structure. Interestingly, the community network can be visualized to identify possible communicating pathways in which perturbations propagates throughout the receptor structure, with communities shown as spheres and communicating edges as rods in a 3D-VMD representation [51]. This analysis was used to explore how a point mutation impacts the network connectivity of the TM domains. The Bio3d package implemented in the R package suit was used to perform the dynamical network analysis [57]. Each Cα atom was used as a communicating node in the network, as we were interested in disclosing backbone correlations along the receptor structure [59]. The network structure relies on the concept of edge betweenness, which measures the number of shortest paths crossing an edge, and the edge with the greatest betweenness is removed on each iteration. The convergence criterion for the network structure is the modularity that represents the probability between the intra- and interconnectivity of communities, with values in the interval 0 to 1. Optimal modularity scores in dynamic correlation network analysis from MD trajectories are typically in the interval from 0.4 to 0.7 [60].

## Results

### Structural integrity of the FSHR in a lipid bilayer of polyunsaturated SDPC phospholipids

#### Structural features of functional I1 and I2 FSHR variants

**Fig 3:**
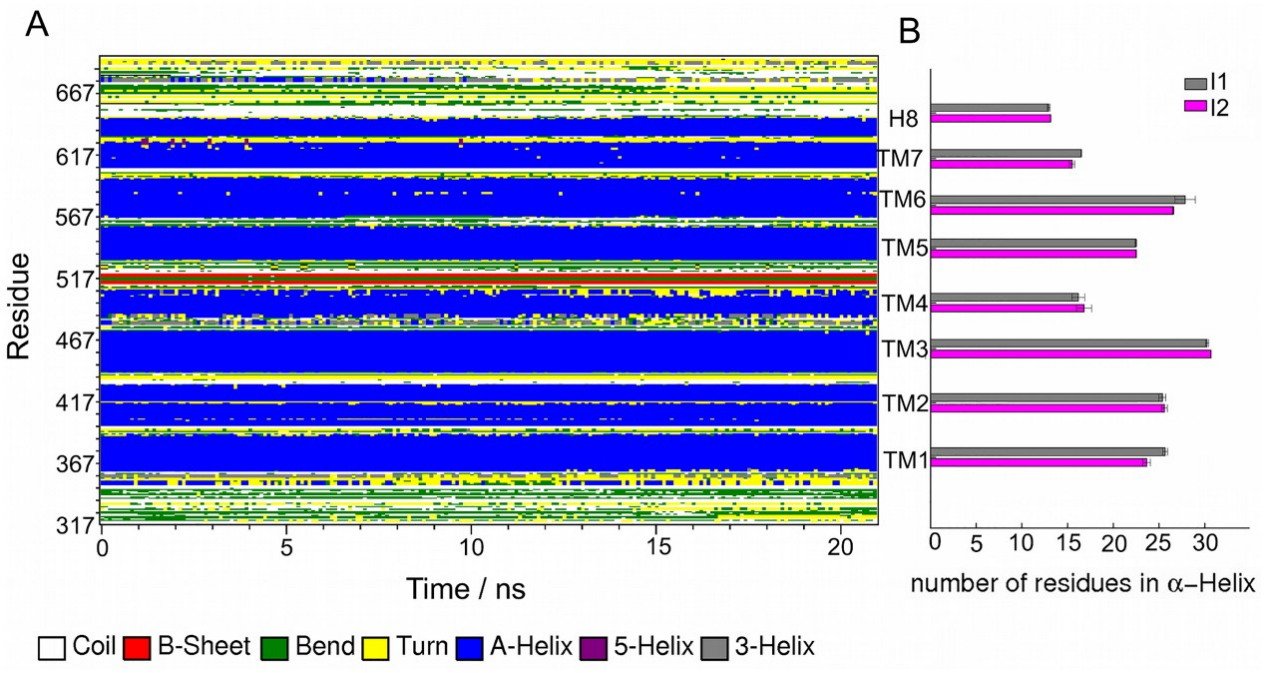
Secondary structure analysis for the FSHR I1 and I2 variants. (A) Secondary structure breakdown of the receptor residues *vs* time for the last 20 ns of trajectory R2 in the I1 variant. Residues forming the TMDs 1 to 7 are represented in blue, β-sheets in red, random coil in white, helix 3 in gray, helix 5 in purple, turns in yellow, and hairpin bends in green. The height of the bands represents the number of residues forming the specific structural pattern. (B) Average number of residues forming α-helix for the TMDs 1–7, and helix 8 domains. TM helix 4 and TM helix 7 were the shortest helical domains with only 15 residues long, while TM helix 3 and TM helix 6 were the largest helical domains with 25–30 residues long. Averages and standard deviations are calculated for 20 ns of the R0-2 trajectories generated for the I1 and I2 variants. See also Fig B in S1 Text.

The TM helical domains of the functional I1 and I2 FSHR variants were well preserved in the lipid bilayer environment as a result of favorable lipid-, water-protein and interhelical interactions (Fig 2A). Because I1 and I2 differ in a single residue at position 680, in the intracellular Ctail, the helical domains were expected to show similar structural features. For example, residues T449, I411, L415, V450, S453, Y530, L537, S589, M585, H615, I588, S589, K608, and V612 were located in the upper half of the receptor (purple surface in Fig 2A), altogether forming a putative binding pocket for drugs [5]. I1 and I2 exposed key extracellular lysines (Fig 2A) such as the conserved K513, as they could interact with acidic residues at the hairpin loop of the hinge region [2, 5]. Palmitoylated cysteines C644 and C646 were well inserted into the lipid bilayer, anchoring helix 8 (H8 in Fig 2A) into the bilayer. To keep the NH_2_-terminus in close interaction with the extracellular domains, we defined a disulfide bond between C338 and C356, and a second bond at the extracellular side, between C442 and C517 [2, 18]. Interhelical water molecules were stable in the internal region of the helix bundle, forming a communicating channel between the extracellular and intracellular regions (Fig 2A). Fig 2B shows the number density for water and lipids across the bilayer for the I1 variant. Density profiles were calculated at 300 K and using three trajectories, R0, R1 and R2, at 310 K for the calculation of standard deviations. Profiles across the bilayer for water, lipid heads (phosphate groups), and lipid tails (methyl groups) of the SDPC molecules allowed us to identify some characteristics of the bilayer topology such as the span of the hydrophobic core, within the interval -10 to 10 Å, and the water-lipid interfaces within the interval 15 to 25 Å for the upper leaflet, and -15 to -25 for the lower leaflet. Interestingly, the water distribution in the interhelical region was similar at 300 K and 310 K, except for a slight density increase in the upper half of the receptor (Fig 2B), which corresponds to the location of the binding pocket. The interhelical water molecules play an important role in the function of membrane receptors; on the one hand, the receptor binding pocket is accessible from the extracellular side and forms a communicating channel toward the intracellular side and, on the other, hydration of side chains promotes the formation of hydrogen bonds that may be important for the conformational dynamics of the receptor [61]. The interhelical water molecules in the hydrophobic core of the bilayer might affect the integrity of the helix domains by turning polar the lipid environment. Therefore, we evaluated the integrity of the TMD helices by a secondary structure analysis as shown in Fig 3A. The bands corresponding to the α-helices showed stable fluctuations, and no significant impact of the hydration levels on the structural integrity of the TM domains was noted. Because the bands width of Fig 3A correspond to the number of residues forming a specific secondary structure pattern, we calculated the number of residues in the α-helix of the TMDs 1 to 7 in the I1 and I2 variants (Fig 3B). The average number of residues forming the α-helix was in fact similar in both variants; TM helix 4 and 7 were the shortest helices with 15 residues, and TM helix 3 was the longest with 30 residues. One additional analysis to assess helix integrity was the calculation of RMSD *vs* simulation time of each TM helix in the I1 and I2 variants (Fig C in S1 Text). TM helices and helix 8, showed fluctuations within 0.5 and 1.5 Å, except for TM helices 1 and 7 of variant I2 at 300 K with RMSD >2 Å. Moderate RMSD values (~2 Å) were observed for TM helices 1, 2, and 6. The overall receptor structure in the I1 and I2 variants was well preserved in our simulation set up in the polyunsaturated lipid environment of SDPC molecules, and fluctuations of the structural domains such as the TM helices were stable in the complexity of the molecular environment of the lipid bilayer. Both, fluctuations and stabilizing interactions form, ultimately, a set of conditions that modulate the receptor function, and disruption interactions in key position may impact the receptor structure, dynamics, and stability, as described below.

#### Structural features of the loss of function M1-3 mutant FSHRs

**Fig 4:**
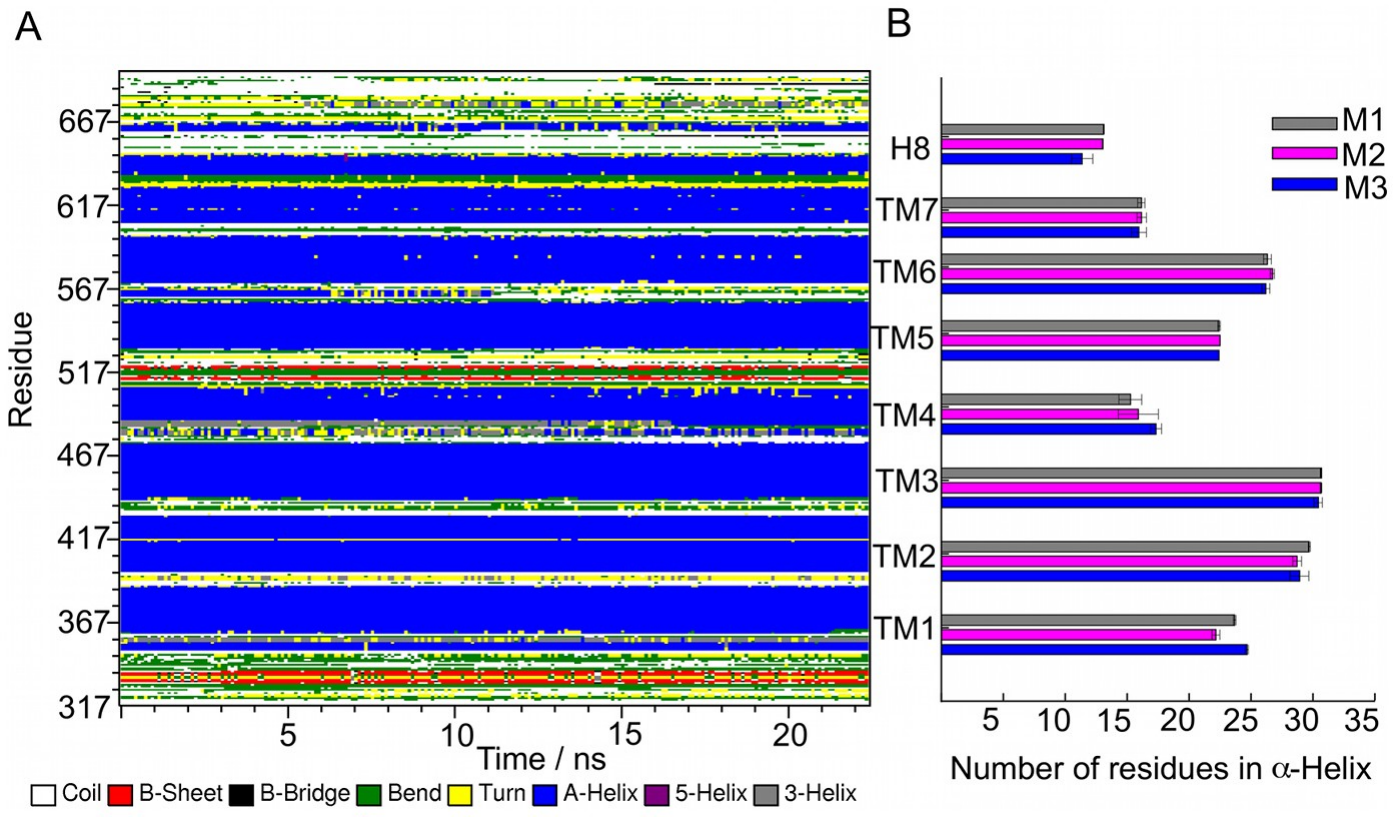
Secondary structure analysis for the FSHR M1-3 mutants. (A) Secondary structure breakdown of the receptor residues *vs* time, for the last 20 ns of trajectory (R2) in the M3 mutant. Residues forming the TMDs 1 to 7 are identified in blue, β-sheets in red, random coil in white, helix-3 in gray, helix-5 in purple, turns in yellow, and hairpin bends in green. The height of the band represents the number of residues forming the specific structural pattern. (B) Average of the number of residues forming α-helix for the TMDs 1–7 and helix 8 domains. TMD helix 4 and TM helix 7 were the shortest helical domains with only 15 residues long, while TM helix 3 and TM helix 6 were the largest helical domains with 20–25 residues long. Averages were calculated for 20 ns of the R0-2 trajectories generated for the M1-3 mutants. TM helix 2 was one turn longer in all mutants when compared to the corresponding TMD in the I1 and I2 variants. See also Fig B in S1 Text.

The mutations introduced in the WT FSHR consisted of replacing the side chain atoms at position 408 from aspartate to alanine in M1, to arginine in M2, and to tyrosine in M3. Residue 408 was located in the upper half of the TM helix 2 (Figs 1C, 1D and 2A). In all mutants, integrity of the TMD was assessed using the same procedure described above for the I1 and I2 variants, namely, a secondary structure analysis and the RMSD for the individual TM helices (Fig D in S1 Text). The calculated RMSD values were within a 1.0 Å– 2.0 Å interval, suggesting stable fluctuations of the TMDs. The bilayer topology and interhelical water molecules were determined by number density profiles across de normal bilayer (Fig E in S1 Text). The results showed that the structural features described above for the I1 and I2 variants, such as putative binding pocket and location of the conserved extracellular lysines, disulfide bonds, and palmitoylated cysteins at helix 8, were also present in the M1-3 mutants (Fig 2A). For comparative purposes, the secondary structure analysis for the M3 mutant is shown in Fig 4A, and the number of residues forming the TM helices in the M1-3 mutants in Fig 4B. In contrast with a previews study that detected a significant disruption of the secondary structure upstream to the TM helix 4 in a model of the D408Y mutant FSHR [20], our analysis revealed an increase in the number of residues in the TM helix 2 from 25.3 ± 0.4 and 25.6 ± 0.3 in the I1 and I2 variants, respectively, to 28.9 ± 0.8 in the M3 mutant, which represents approximately one-turn difference. The additional turn of TM helix 2 was also observed in the M1 and M2 mutant FSHRs (Fig 4B). Thus, disordering of TM helix 2 due to the D408Y mutation previously found [20] was not replicated in our secondary structure analysis.

The interhelical hydration level and the trajectory lengths for all systems are summarized in Table 1. Approximately, fifty water molecules were found in the interhelical region, with a minimum of 37 molecules for the M3 mutant at 310 K and a maximum of 73 molecules for the I2 variant at 300 K. The relative variability of the hydration level had no impact on the stability of the TM helices in any of the FSHR mutants.

**Table 1:** Trajectory length and hydration level in the interhelical region. The number of water molecules in the hydrophobic core was calculated by integrating the water density profiles from -10 to 10 Å in the hydrophobic core of the bilayer.

System	300K		310K	
	Trajectory length ns^e^	Number of interhelical water Molecules^f^	Trajectory length ns	Number of interhelical water Molecules
I1				
R0^a^	70.654	53.18–54.35	51.516	41.81–42.51
R1^b^			21	47.42–48.41
R2^c^			21	46.80–47.54
R3^d^			21	52.46–53.25
I2				
R0	64.308	71.22–72.81	52.5	52.83–53.92
R1			22.5	52.33–53.74
R2			22.5	54.87–56.16
M1				
R0	66.664	45.8–46.6	52.074	42.87–43.63
R1			22.47	41.99–42.93
R2			22.5	44.24–45.20
M2				
R0	63.121	43.49–44.39	52.074	47.01–48.05
R1			22.5	50.05–51.20
R2			22.5	42.76–43.58
M3				
R0	70.805	55.20–56.58	52.734	36.89–37.58
R1			22.5	38.51–39.45
R2			22.343	37.78–38.51

^a^Main trajectory;

^b^Trajectory initialized at ~30 ns of R0;

^c^Trajectory initialized at the end of R0;

^d^Trajectory initialized at 37 ns of R0.

^e^Simulation length trajectories completed according to availability of supercomputing resources.

^f^Integration of the number density profile in the interval -10 Å to 10 Å in volume elements dz*(A_average_ +/-).

#### Interhelical interactions of the side chains of residue 408

**Fig 5:**
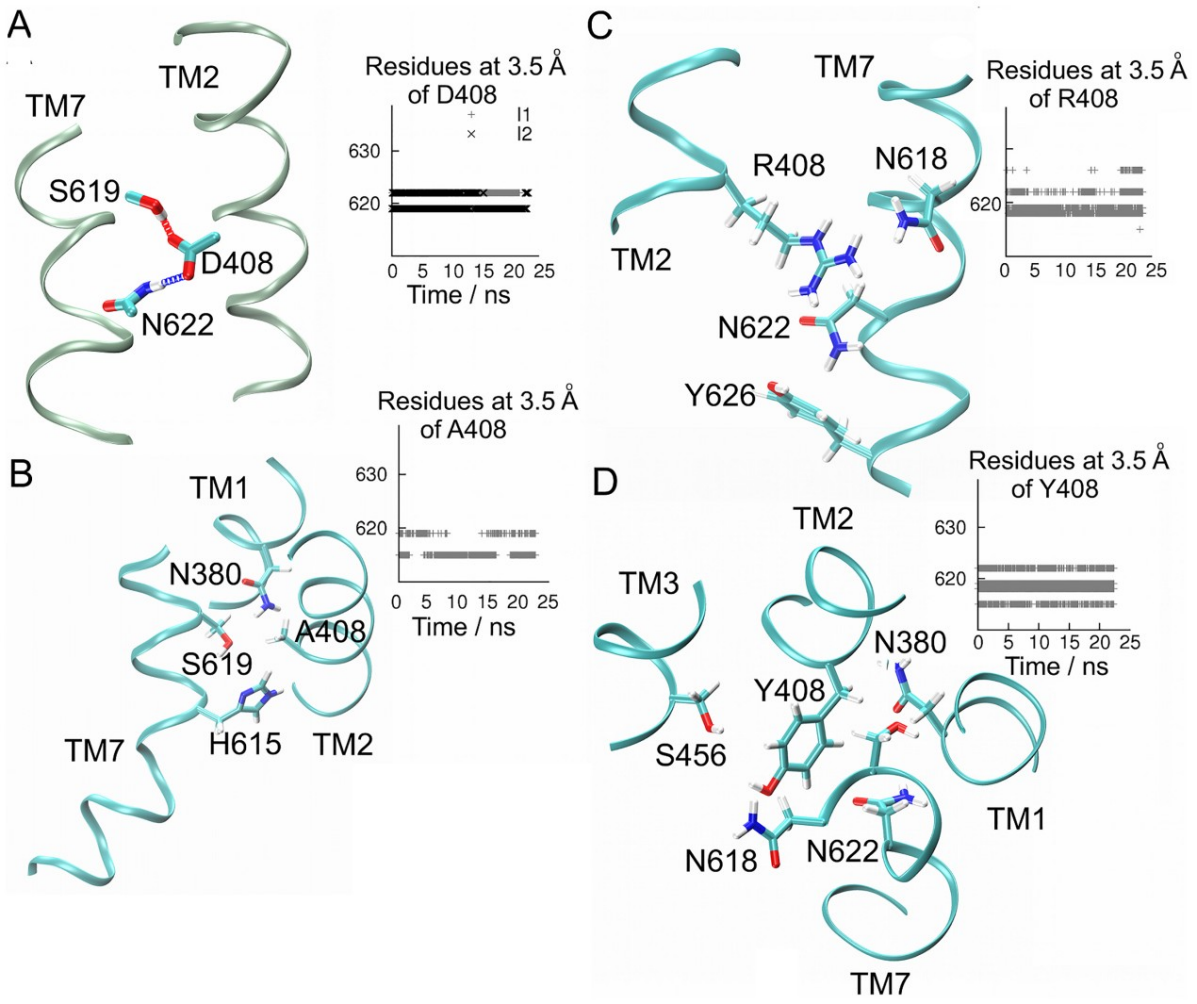
H-bonds interactions of side chain atoms of D408 in I1 and I2 variants, and contact maps in mutants M1-3. For clarity only small fragments of TM2 and TM7 are depicted as ribbons. Side chain atoms are depicted in licorice and the following color code: C-cyan, O-red, N-blue, H-white. (A) H-bonds (dashed lines) between D408 at TM2, and S619 and N622 at TM7. (B) Contact map for alanine 408 in M1 mutant. Represented TM1, TM2 and TM7, and side chain atoms of A408, N380, H615, and S619. (C) Contact map for arginine 408 in M2 mutant. Represented TM2 and TM7, and side chain atoms of N618, N622 and Y626. (D) Contact map for tyrosine 408 in mutant M3. Represented TM1-3 and TM7, and side chain atoms of N380, S456, N618 and N622. The inset plots in panels A-D show all contacts detected as function of simulation time for the residue at position 408. Contacts of side chain atoms at 408 were different upon mutation of aspartate. Data collected for 20 ns of trajectory R1 at 310 K. See also Fig G in S1 Text.

Interhelical interactions were disrupted upon mutation of aspartate 408. In I1 and I2 variants, direct hydrogen bonds between the carboxylate group and hydroxyl groups of S619 and the amide group of N622 of TM7 were detected (Fig 5A). The interaction between helices TM2 and TM7 was broken in the M1 mutant because of the aliphatic side chain of alanine; in M2 the large side chain of arginine was close to N618, N622 and Y626, without forming direct hydrogen bonds; finally, in M3 the tyrosine ring approached to N380 of TM1, S456 of TM3, and N618 and N622 of TM7. None of the mutants were able to form interhelical hydrogen bonds due to the replacement of the carboxylate group of D408 with side chains incompatible for hydrogen bonding (Fig 5). We then examined the impact of these interactions on the conformational dynamics of the receptor backbone in terms of fluctuations of Cα atoms, analysis of principal components, correlations, and formation of dynamical networks communities as a benchmark to measure the extent in which a point mutation may promote larger conformational changes.

### Conformational dynamics of the FSHR backbone in functional and dysfunctional phenotypes

**Fig 6:**
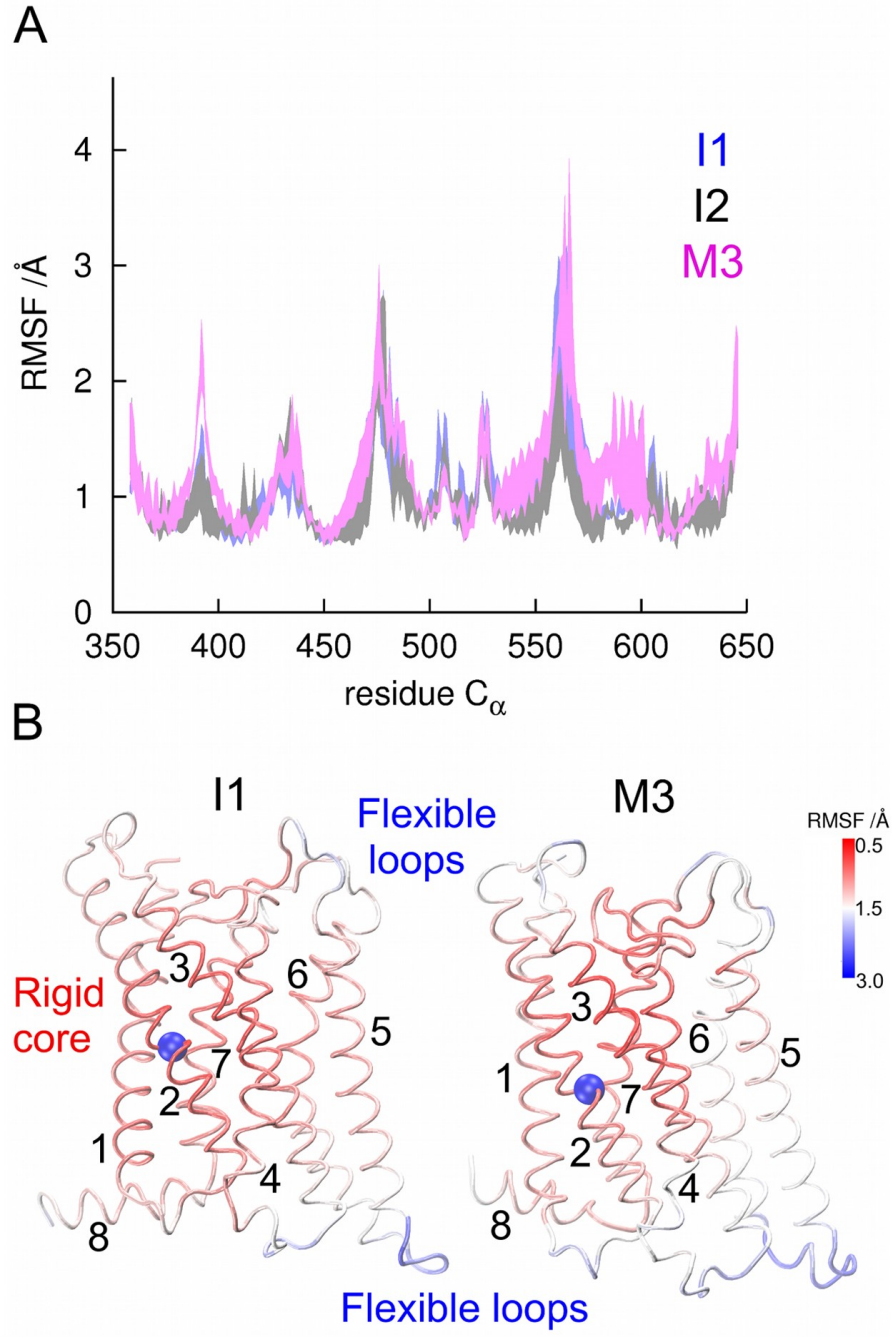
Root mean square fluctuations (RMSF) for Cα atoms of residues 358 to 646 (TMD). (A) RMSF of I1 and I2 variants, and the M3 mutant, at 310 K. Line widths correspond to standard deviations calculated for trajectories R0-2 of each system. Larger RMSF values identify flexible regions of the receptor, and lower values rigid regions. (B) Structure of the seven TM helices (1–7) and helix 8 colored in a red-white-blue color scale according with the RMSF values for those in the interval 0.5 to 3.0 Å. Intracellular regions were the most flexible including intracellular loops connecting helix 1 and helix 2, helix 3 and helix 4, and helix 5 and helix 6, and the intracellular halves of TM helix 5 and 6. Significant increase of fluctuations was noted in the M3 mutant from TM helix 5 to helix 8.

Fluctuations along the backbone were calculated by means of the RMSF of Cα atoms as a measure of the amplitude of the atom motion relative to its average position. Fig 6 displays a comparison of the RMSF for the I1 and I2 variants *vs* M3 for runs R0-2 at 310 K, with the line widths defined by the standard deviation (± ϭ). From these data, flexible and rigid regions were identified; for example, loops connecting TM helices showed larger RMSF values (>2 Å), whereas rigid helices showed lower RMSF values (<1 Å). Intracellular regions were the most flexible in the I1 and I2 variants. The rigid core encompasses TM helices 1 to 3, and the flexible regions TM helices 4 to 7 (Fig 6B). The second intracellular loop (IL2) connecting TM helices 3 and 4, peaks at L477, and is located in a interesting location next to the highly dynamics intracellular region of TM helices 5 and 6, which have been described as flexible regions in other GPCR [35]. In the M3 mutant flexibility increased significantly when compared to the I1 and I2 variants, in a larger extent from TM helix 5 to helix 8 (Fig 6A). Fluctuations calculated for the FSHR variants and mutants showed remarkable differences between the functional and dysfunctional variants (Fig F in S1 Text), that could hint some important differences to understand the abnormal expression and activation found in FSHR likely induced by disruption of specific interhelical interactions at position 408.

The conformational dynamics of the TM domains of the FSHR model was further explored by a principal component analysis (PCA) of the variance-covariance matrices. We focused on the first principal component (PC1) because it represents the largest contribution to the total fluctuation. By projecting the simulation trajectories over PC1, collective motions of the TM domains were identified. For trajectories R0 and R2, spanning ~70 ns of simulation time for each phenotype, the cosine content (CC) was calculated to evaluate the contribution of random thermal fluctuations over the PC1 axis. CC values close to 1 indicate random diffusion, which could be used as a parameter for evaluation of convergence of fluctuations [56]. We found relatively high random fluctuations according to the CC values of the PC1: 0.89 in I1, 0.82 in I2, 0.74 in M1, 0.90 in M2, and 0.44 in M3. Thus, thermal fluctuations were part of the dynamics detected along the PC1 axis, suggesting that the receptor backbone fluctuations were essentially slight deviations from an average structure stabilized by the molecular environment of the lipid bilayer, water molecules in the interhelical region, and receptor intramolecular interactions. In this setting, the PCA analysis served to identify differences in the global dynamics that were likely induced by point mutations in a similar background of thermal fluctuations. The global motion of the TM domains in the I1 variant, which highlights some comparative differences between I1 and the FSHR I2 variant and M1-3 mutants are next described.

**Fig 7:**
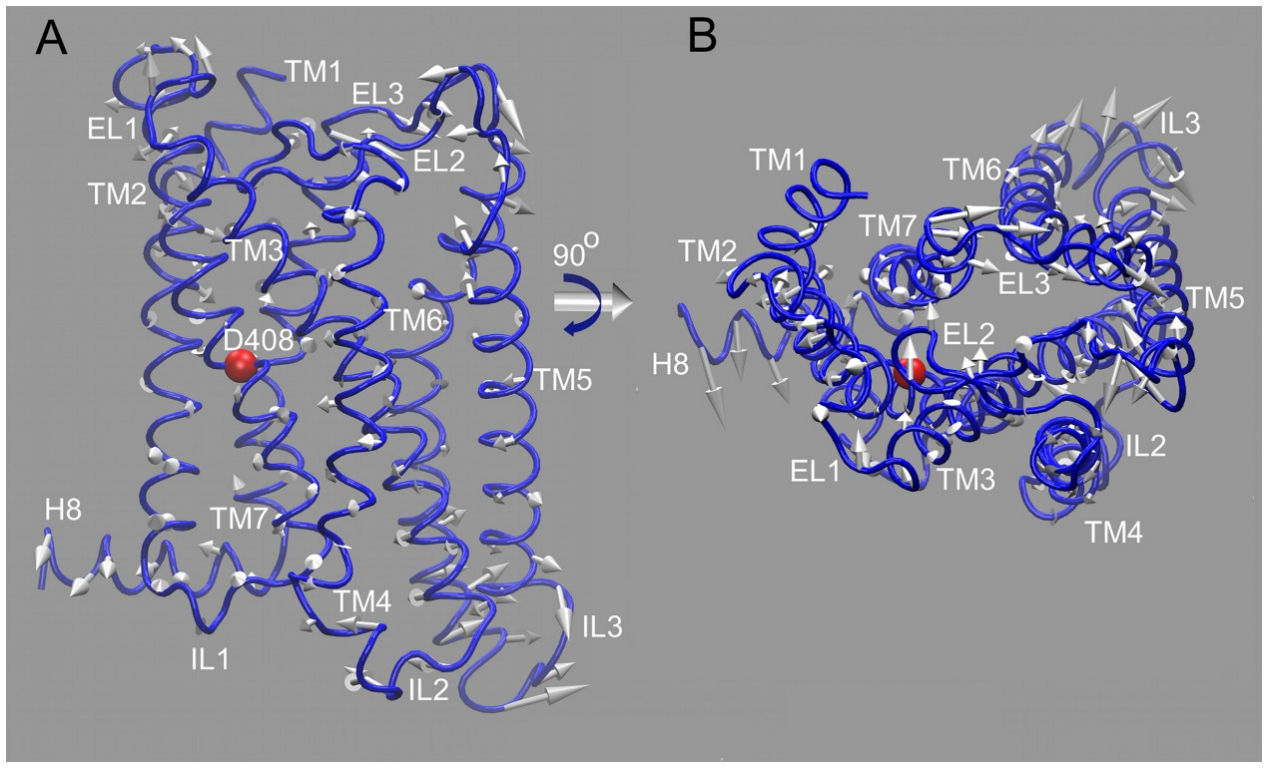
Projection simulation trajectory of the I1 variant over the first principal component axis. (A) Side view of the TM domains showing the initial conformation of the I1 variant (blue ribbons). D408 (red sphere) is located in TM helix 2. White arrows represent the motion of the Cα atoms and define both direction and distance spanned by the motion along the PC1 axis. (B) Intracellular view of the I1 variant. EL3 moved toward TM helix 6, while helix 7 move upward to the extracellular side. TM helices 2–4 formed a rigid core, and intracellular loops IL2 and IL3 were flexible regions moving in opposite directions.

Visualization of the motion projected over the PC1 axis in the I1 variant confirmed the flexibility of intracellular domains found by the RMSF calculations (Fig 6). In fact, position D408 seems to be important for stabilizing specific interactions among the TM helices, as suggested by the motion over PC1, where TM helix 2–4 formed a rigid region in a flexible context such as the up-dawn motion of TM helix 7, the seesaw motion of TM helix 1 and 5, and the back and forth contortion of TM helix 6 (Fig 7; S1 Movie). Helix 8 moved in concert with TM helix 7; specifically, the upward motion of the TM helix was linked to a back and forth motion of helix 8 in the bilayer plane (Fig 7B). Intracellular loops were very dynamic, particularly the loop connecting the TM helices 3 and 4 (IL2), which moved in concert to the loop connecting TM helices 5 and 6 (IL3); the former moved inwards to the helix bundle whereas the latter moved away from the helix bundle, in perpendicular direction (S1 Movie). At the extracellular side, the loop between TM helix 6 and 7 (EL3) moved along with its connecting helices, i.e. the lift of TM helix 7 and the backwards contortion of helix 6 correlated with the motion of EL3 from helix 7 to helix 6 (Fig 7B). EL3 also correlated with the motion of the EL2 between TM helix 4 and 5 (EL2; Fig 7B). In comparison to I1, the I2 variant showed smaller amplitudes (Fig H in S1 Text). Interestingly, the motion of helix 8 moved in direction to helix 7 causing disruption of the last turn in the TM α-helix (Fig H in S1 Text). In I2 the rigid core formed by TM helices 2–4 was present, with a slight contortion of TM helix 2 (S2 Movie). In contrast to the motion observed in the I1 variant, the loops of the M1 mutant showed almost no motion along the PC1 axis; the main backbone motion was a lift of the TM helix 6 (S3 Movie). In the M2 mutant, the backbone motion showed two anti-correlated regions. TM helices 1, 6, 7, and helix 8 moved upwards while TM helices 4 and 5 moved downwards (S4 Movie; see also Fig I in S1 Text). TM helices 2 and 3 still formed a rigid core, only TM helix 3 was straight at the end of the motion (S5 Movie). In M3, the amplitude backbone motion was smaller than that in the I1 variant (S6 Movie; see also Fig J in S1 Text). The main backbone motion was a lift of TM helix 6 and helix 8, a contortion of TM helix 5, and the seesaw motion of TM helix 1 and 7. In summary, the first principal component allowed us to compare motions of the backbone atoms along the PCI axis, in a similar background of thermal fluctuations. The M1 and M3 were the phenotypes with the smallest overall fluctuations along the PC1 axis, which might be an indication that interactions induced by charged side chains such as aspartate or arginine induced important interactions in the TM domains.

#### Dynamical network analysis for the FSHR variants and mutants

**Fig 8:**
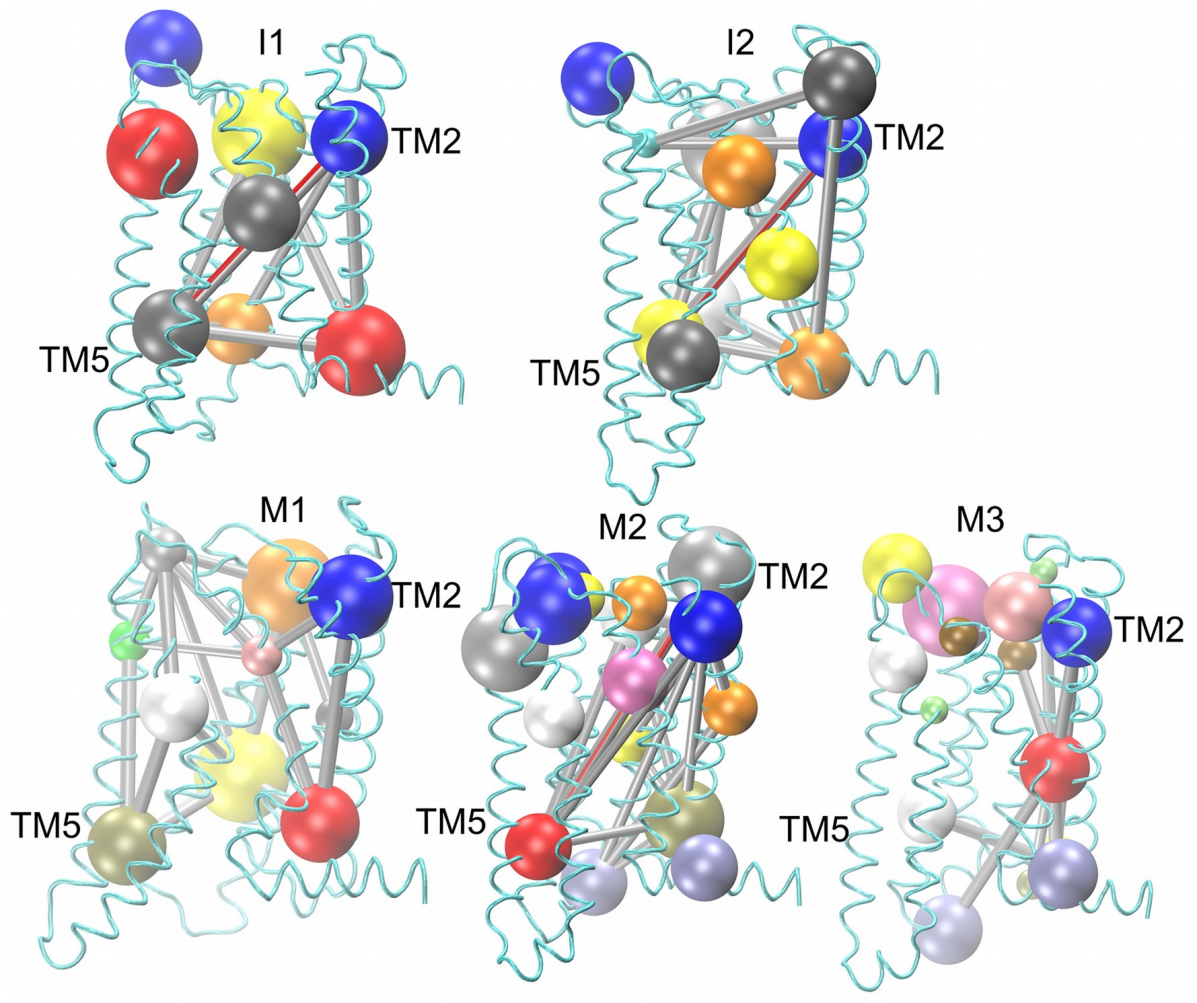
Dynamical network analysis of the FSHR variants I1 and I2, and M1-3 mutants. Each sphere represents a community including at least 10 Cα atoms. Edges connecting communities represent communicating pathways among the TMDs. Highlighted in red the edge connectivity between communities of TM helix 2 and TM helix 5 in I1, I2 and M2. The network structure of M1 and M3 showed differences in the connectivity relative to the functional I1 and I2 phenotypes and the M2 mutant. The FSHR backbone is depicted in cyan ribbons to provide the context of the location of the network communities.

Cross correlations of the Cα fluctuations were calculated from the normalized variance-covariance matrix. The correlation coefficients stored in the dynamical cross correlation matrix (DCCM) were used to perform a community network analysis over the full length of trajectories at 300 K and 310 K, for time frames stored every 100 ps, in order to include all the variability of the backbone sampled in our simulations. Residues highly correlated (cutoff-c_ij_ = 0.7) were identified as communities from the DCCM calculation (Fig K in S1 Text). In Fig 8 the communities (spheres) and edges (rods) connecting communities are shown. Only communities including at least 10 Cα atoms were represented. Communities at the upper and lower segments of the TM helices were consistently found in all phenotypes; in particular, we focused on the communities in the upper segment of the TM helix 2, and the lower segment of TM helix 5. On the one hand, the TM helix 2 community shown in Fig 8 was in a location close to D408 and the rigid core identified in the PCA analysis (Fig 7), which also coincide with the location of the putative binding site (Fig 2A). On the other hand, community of the TM helix 5 shown in Fig 8 was located in a very dynamic region according to RMSF and PCA analysis (Figs 6 and 7). Interestingly, the TM helix 2 and TM helix 5 were edge connected in I1, I2 and M2 phenotypes, but no edge connection was found in M1 and M3 (Fig 8). The difference in the network connectivity of phenotypes with a charged side chains at position 408 (such as I1, I2, and M2) and phenotypes with polar or non-polar (such as M1 and M3) suggests that interactions promoted by electrostatic interactions in the interhelical region play a role in the global dynamics of the receptor structure. Therefore, it is expected that mutants M1 and M3 behave in a similar fashion regarding expression and function, as shown in Fig 1A and 1B, and Fig A in S1 Text, while the M2 mutant could share similarities in expression to I1 and I2 (Fig A in S1 Text).

## Discussion

In the present study, we set up a computational approach to develop a model for the TMDs of the FSHR, combining modeling and MD simulation methods using the all-atom force field approximation. The TMD of the FSHR variants and mutants were stable in the bilayer topology generated by polyunsaturated SDPC lipid molecules, including the bulk solvent region, the water-lipid interfaces, and the hydrophobic core. Our computational implementation provided a working FSHR model to unveil structural features of the TMD as well as conformational dynamics at room and physiological temperatures that could be relevant for targeting the glycoprotein hormone family of GPCRs for drug discovery [62]. The computational protocol we set up consisted of the following stages: a. Generation of initial receptor coordinates by the GPCR-I-Tasser [12]; b. Insertion of the model in a lipid environment of polyunsaturated lipid molecules; c. Addition of ions for system neutrality and solvent water molecules for hydration of the protein loops, NH_2_- and COOH-terminus, and lipid heads; d. Generation of a simulation trajectory at physiological temperature; and f. Analysis of the conformational dynamics in terms of fluctuations of secondary structure, principal components, or dynamical network analysis. Our computational approach, addressed to understand the structure and dynamics of a prototype member of the glycoprotein hormone receptor family, may be implemented for the study of other related receptors, such as the TSHR or the LHCGR, to analyze some physiological aspects at the molecular level [5].

The FSHR model of the TMDs described herein was sensitive to point mutations at the middle of the TM helix 2, confirming the key role of D408 on the interhelical environment. The electrostatics of the side chain of aspartate (carboxyl group) induces interactions with internal water molecules and other charged or polar amino acid side chains. Contact maps for D408 showed interactions with S619 and N622 (TM helix 7) in the functional I1 and I2 variants; in fact, interactions between TM2 and TM7 helices have been described for family A (rhodopsin-like) of GPCRs [62]. In the case of the M3 mutant, which leads to hypergonadotropic primary amenorrhea [20], we found that replacement of aspartate with tyrosine at this position led to disruption of the interactions between TM helix 2 and helix 7 detected in the functional variants, with Y408 showing contacts with N380 of TM helix 1, S456 at TM helix 4, C584 at TM helix 6, and H615, N618, and N622 at TM helix 7, not exhibited by the WT I1 variant. The interhelical interactions disrupted in Y408 impacted on the receptor dynamics by reducing the conformational variability at the backbone level, which could be a feature recognized by the quality control system of the cell, thereby leading to intracellular trapping of the mutant receptor [21], a situation shared by M1. Map contacts in M1 and M2 mutants also evidenced the influence of the side chain atoms at position 408: a. Alanine side chain in M1 almost lost interaction with S619 at TM helix 7; and b. The large side chain of the arginine residue in M2 was rather promiscuous, showing contacts with N380 at TM helix 1, C584 at TM helix 6, and N618 and N622 at TM helix 7. Accordingly, local interactions and electrostatics of the side chain atoms provoked distinctly different conformational dynamics of the FSHR according to the fluctuation analysis performed (such as the PCA) and the formation of highly correlated groups of atoms (communities network) dynamically interconnected. Global fluctuations detected by PCA revealed that the first principal component of the I1, I2 variants and M2 mutant, showed larger concerted motions than those of M1 and M3 mutants. It is possible that these similarities may be due to the strong electrostatics of the arginine side chain atoms, in contrast to the alanine and tyrosine side chain atoms of the M1 and M3 mutants.

In a network community analysis of highly correlated regions, networks of the I1 and I2 variants and M2 mutant showed some similar features. For example, the flexible intracellular halves of TM helix 5 and 6 were connected directly or indirectly to TM helix 2, where D408 was indeed located (Fig 8). This observation confirms the key role of D408 in the structure and the conformational dynamics of the FSHR; on the one hand its location in the putative binding pocket and, on the other, its role on communication pathways for conformational changes. Our results suggest that the residue at position 408 impacts on the conformational dynamics due to specific protein interactions that could promote correlations throughout to the whole receptor structure.

Variants at position 680 (I1 and I2) have been described as common functional phenotypes of the FSHR in humans [23]. In assisted reproduction, variant I2 shows lower response to ovarian stimulation, among other differences in biological function [26]. In the cell machinery, the recognition of the FSHR variants may involve epitopes or motifs that induce different cellular responses [2]. Because of its location, having asparagine or serine at positions 680 likely triggers distinct internalization dynamics and signaling processes associated with the Ctail rather than to the structure of the TM domains [24, 25, 63]. In fact, variants I1 and I2 showed structural integrity of the TM domains with similar residue length of the α-helix structure and TM helix 2-TM helix 5 network community connectivity. The intricate balance between flexible or rigid regions could impact the overall conformational dynamics, an aspect that somehow modulates the physiological function of the receptor, such as that of FSHR variants with asparagine/serine at position 680.

## Conclusions

Current computational methods provide a set of strategies to uncover the impact of small differences in a few atoms (as occurs by point mutations), on the conformational dynamics of a membrane receptor in the complexity of the lipid bilayer environment. Our benchmarks on simulation trajectories in the ~100 ns time scale, including 130 thousand atoms, suggest that the specific interactions in the interhelical region impact the backbone conformational dynamics of the FSHR. Non-polar or polar side chain residues at 408, such as alanine or tyrosine, disrupted the concerted backbone motions. More specifically, H-bonds promoted by aspartate at 408 between TM helix 2 and 7 seem to be essential to preserve the receptor functionality. The strategy we used to test the relevance of point mutations in the transmembrane domain of the FSHR could help to understand the impact of point mutations on other members of the glycoprotein receptor family of GPCR.

## Supporting information

S1 Text Experimental data for the evaluation of the expression and activation of the FSRH variants and mutants. Computational data obtained for evaluation of the structure and conformational dynamics of the FSHR variants and mutants. **Table A**. Oligonucleotide primers used to construct the FSHR S680 variant and mutant receptors cDNAs. **Fig A**. Experimental data for functional identification of the FSHR M1 and 2 mutants. **Fig B**. Secondary structure analysis for FSHR I1 and I2 variants and M1-3 mutants **Fig C**. RMSD calculation for the TM helices in the FSHR variants I1 and I2 **Fig D**. RMSD calculation for the TM helices in the FSHR mutants M1-M3 **Fig E**. Number density profiles across the bilayer normal for water, phosphate, and methyl groups of the lipid molecules **Fig F**. Root mean square fluctuations (RMSF) for the Cα atoms of M1 and M2 mutants. **Fig G** Map contacts for the I1 variant and the M1-3 mutants at 300K and 310K. **Fig H**. Motion of the TM domains along the PC1 for the I2 FSHR variant. **Fig I**. Motion of the TM domains along the PC1 for the M2 FSHR mutant **Fig J** Motion of the TM domains along the PC1 for the M3 FSHR mutant. **Fig K**. Chart for dynamical cross correlation matrices for the FSHR phenotypes. **Table B**. Number of atoms and transversal box area for each system **Table C**. Parameters for local regression planes for the motion of Ca atoms projected over the first principal component. **Fig L**. Parameterized plane for R0-2 of the first Cα in the S12 Table.
(DOCX)

S1 Movie Motion of fluctuations projected over the first principal component axis in the I1 vairant. (MPG)

S2 Movie Motion of fluctuations projected over the first principal component axis in the I2 vairant. (MPG)

S3 Movie Motion of fluctuations projected over the first principal component axis in the M1 mutant. (MPG)

S4 Movie Motion of fluctuations projected over the first principal component axis in the M2 mutant. (MPG)

S5 Movie Intracellular view of the fluctuations projected over the first principal component axis in the M2 mutant. (MPG)

S6 Movie Motion of the fluctuations projected over the first principal component axis in the M3 mutant. (MPG)

S7 Movie Intracellular view of the fluctuations projected over the first principal component axis in the M3 mutant. (MPG)
