# Supplementary material for: Molecular dynamics simulation of the follicle-stimulating hormone receptor. Understanding the conformational dynamics of receptor variants at positions N680 and D408 from in silico analysis
Source: PLoS One. 2018 Nov 21;13(11):e0207526. doi: 10.1371/journal.pone.0207526 (PMC6248991; doi:10.1371/journal.pone.0207526)
Supplement: S1 Text — Computational data obtained for evaluation of the structure and conformational dynamics of the FSHR variants and mutants. Table A. Oligonucleotide primers used to construct the FSHR S680 variant and mutant receptors cDNAs. Fig A. Experimental data for functional identification of the FSHR M1 and 2 mutants. Fig B. Secondary structure analysis for FSHR I1 and I2 variants and M1-3 mutants Fig C. RMSD calculation for the TM helices in the FSHR variants I1 and I2 Fig D. RMSD calculation for the TM helices in the FSHR mutants M1-M3 Fig E. Number density profiles across the bilayer normal for water, phosphate, and methyl groups of the lipid molecules Fig F. Root mean square fluctuations (RMSF) for the Cα atoms of M1 and M2 mutants. Fig G Map contacts for the I1 variant and the M1-3 mutants at 300K and 310K. Fig H. Motion of the TM domains along the PC1 for the I2 FSHR variant. Fig I. Motion of the TM domains along the PC1 for the M2 FSHR mutant Fig J Motion of the TM domains along the PC1 for the M3 FSHR mutant. Fig K. Chart for dynamical cross correlation matrices for the FSHR phenotypes. Table B. Number of atoms and transversal box area for each system Table C. Parameters for local regression planes for the motion of Ca atoms projected over the first principal component. Fig L. Parameterized plane for R0-2 of the first Cα in the S12 Table. (DOCX) [file pone.0207526.s001.docx]

**Molecular dynamics simulation of the follicle-stimulating hormone receptor. Understanding the conformational dynamics of receptor variants at positions D408 and N680 from *in silico* analysis.**

Eduardo Jardón-Valadez^1*^, Derik Castillo-Guajardo^2^, Iván Martínez-Luis^3^, Rubén Gutiérrez-Sagal, Teresa Zariñán^3^, and Alfredo Ulloa-Aguirre^3^

**Table A.** **Oligonucleotide primers used to construct the FSHR S680 variant and mutant receptors fragments cDNAs**.

| FSHR | Oligonucleotides | Sequence |
| --- | --- | --- |
| S680 | Sense  Antisense | 5’-TGGTTCCACTTACATACTTG-3’  5’-GAGGTGACTCTGGGAGC-3’ |
| A408 | Sense  Antisense | 5’-GCTCTCTGCATTGGAATCTAC-3’  5’-AGCAAAGGCCAGGTTGCA-3’ |
| R408 | Sense  Antisense | 5’-AGACTCTGCATTGGAATCTAC-3’  5’-AGCAAAGGCCAGGTTGCA-3’ |
| Y408 | Sense  Antisense | 5’-TATCTCTGCATTGGAA-3’  5’-AGCAAAGGCCAGGTTG-3’ |


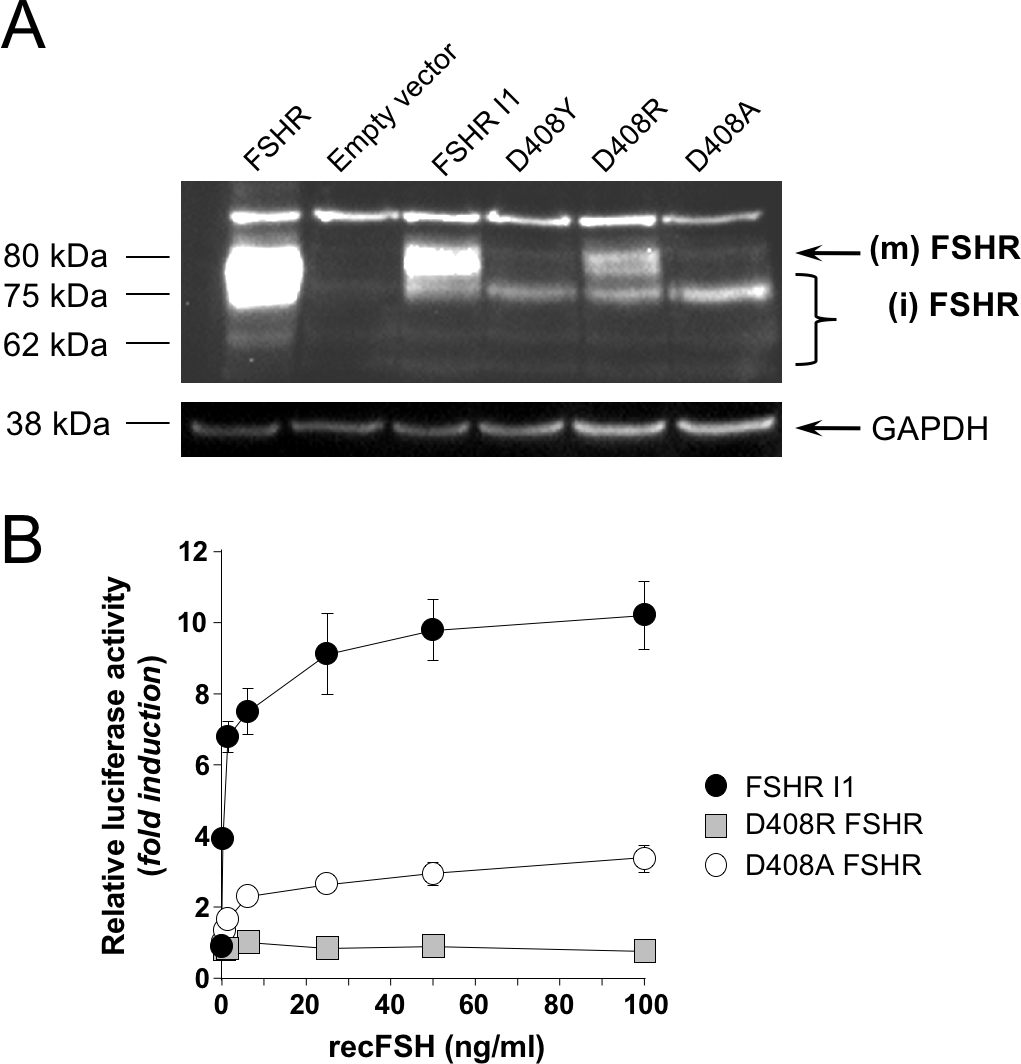


**Fig A. Experimental data for functional identification of the FSHR M1 and 2 mutants.** (A) Western blot of FSHR protein variant I1 (FSHR I1) and mutants M1-3 (D408A, D408R, and D408Y, respectively). The blot shows the migration of FSHRs from protein extracts of HEK293 cells transiently transfected with the WT or mutant FSHR cDNAs inserted in the pSG5 vector. The first lane from left to right, shows the migration of the WT FSHR I1 variant from HEK293 cells stably expressing the receptor. The immunoblot shows that mutant D408R is present as both mature [plasma membrane expressed, kDa ~80; (m)] and immature [intracellular, kDa ≤ 75; (i)] forms, albeit the expression of the mature form is subnormal compared with that of the WT FSHR I1 (third lane). In the case of the D408A mutant the expression of the mature form is marginal and that of the immature form predominates. (B**)** Recombinant FSH-stimulated intracellular signalling of the WT FSHR I1 and the M1 and M2 mutants, as assessed by a reporter gene assay in HEK293 cells transiently cotransfected with the WT or mutant FSHRs and the cAMP-sensitive pSOMLuc reporter plasmid. The results showed that the WT FSHR induced a robust dose-dependent response in luciferase activity, whereas the D408A and D408R mutants showed either markedly reduced or marginal responses to FSH stimulation, respectively (areas under the curve of fold induction in luciferace activity: WT FSHR I1= 934 ± 149; D408A= 286 ± 11; D408R= 87 ± 3; p<0.05 WT *vs* M1 and M2 FSHR and M1 *vs* M2 FSHR, one-way ANOVA followed by Student’s *t*-test).


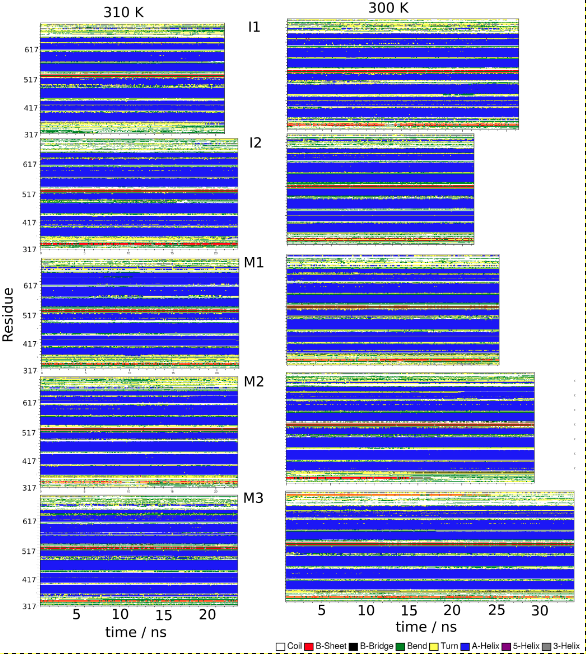


**Fig B. Secondary structure analysis for all phenotypes of the FSHR.** All TM helices were stable and the beta-sheet motif at the extracellular loops (typical of family A of GPCRs) was identified. Analysis performed over the second half of the simulation trajectory at 300 K, and R0 at 310K.


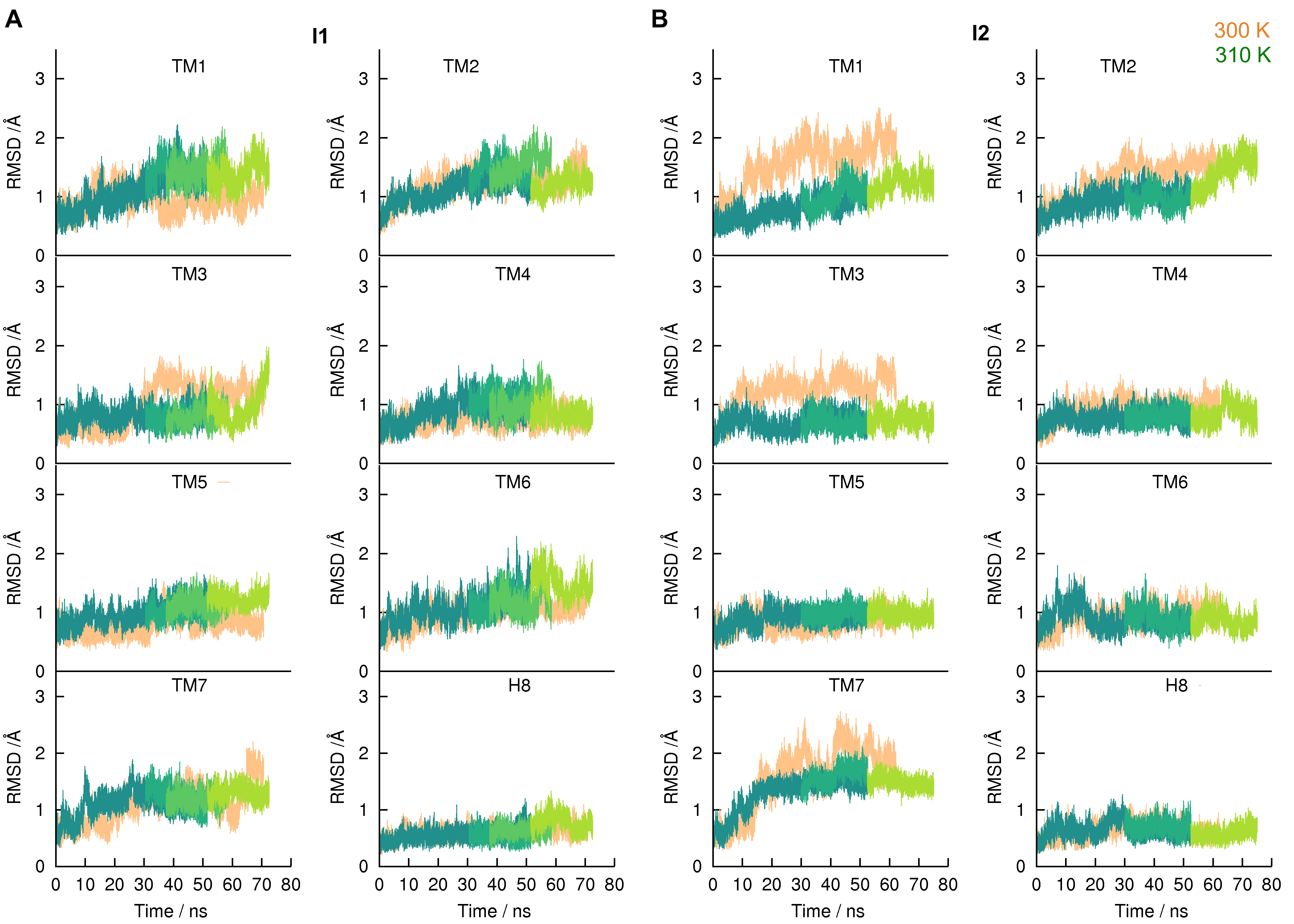


**Fig C. Time series for the RMSD of Cα atoms of helices TM1-7, and helix 8, at 310 K (green solid lines) and 300 K (light orange solid lines).** Dark to light green tones were used to identify analysis for the R0-2 replicas: R0 dark green, R1 green, and R2 light green. **(**A) Calculations for the I1 variant including a R3 repeat using 37 ns as initial coordinates and velocities. (B) Calculations for the I2 variant. TM helices 4, 5, and 6, and helix 8 were stable, and preserved their conformation at 300 and 310 K, in both I1 and I2 variants. TM helices 1, 2, 3, and 7 showed larger displacements at, for example, TM helix 1 and TM helix 3 of variant I2 at 300 K.


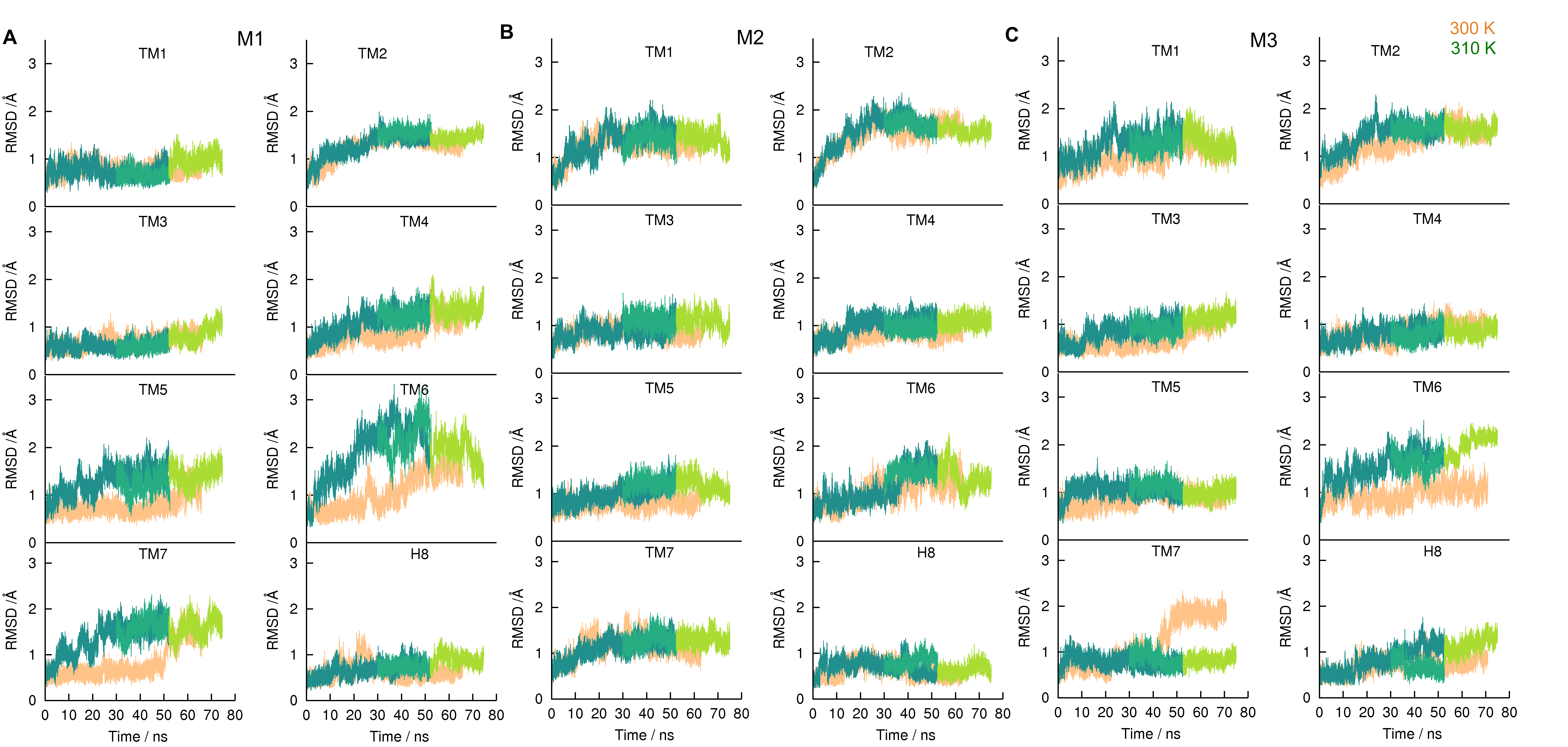


**Fig D. Time series for the RMSD of Cα atoms of helices TM1-7, and helix 8, at 310 K (green solid lines), and 300K (light orange solid lines).** Dark to light green tones were used to identify analysis for the R0-2 replicas: R0 dark green, R1 green, and R2 light green. **A.** Calculations for mutant M1. **B.** Calculations for mutant M2. **C.** Calculations for mutant M3. Moderate displacements (1Å<RMSD<2Å) were observed in all TM domains, except for TM helix 6 of M1 at 310 K where large values of RMSD were calculated (RMSD>2.5 Å). In TM helix 7 of M1 and M3 at 300 K, RMSD values increased from 1Å to 2Å, likely due to a disruption of the side chain interactions between TM helix 2 and helix 7.

**
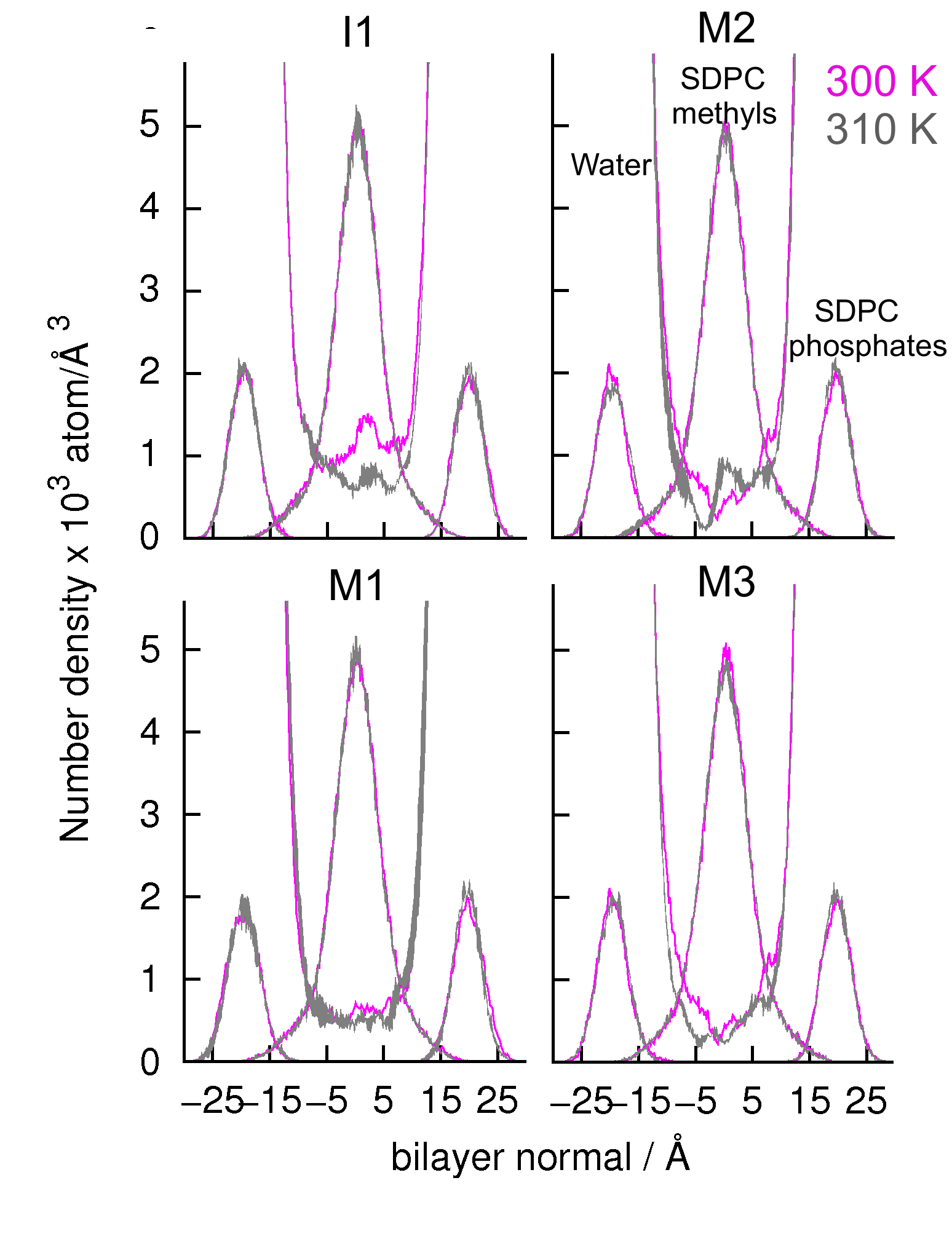
**

**Fig E. Number density profiles across the bilayer normal for water, phosphate, and methyl groups of the lipid molecules.** The width of the gray lines represent the error bars estimated for the R0-2 at 310 K. For comparison, density profiles at 300 K were calculated (magenta solid lines). The hydrophobic core of the bilayer was defined using the distribution of the SDPC terminal methyl groups, and interfaces were located by the distribution of the SDPC phosphate groups.

**Fig F. Root mean square fluctuations (RMSF)** f**or Cα atoms of M1 (top) and M2 (bottom) mutants.** Minimum wells represent TM helical domains, and peaks represent flexible loops. Fluctuations of the backbone were reduced in comparison to I1 and I2 variants. Data collected for trajectories R0-2 at 310 K, and for the trajectory of the M1-3 mutants at 300 K.


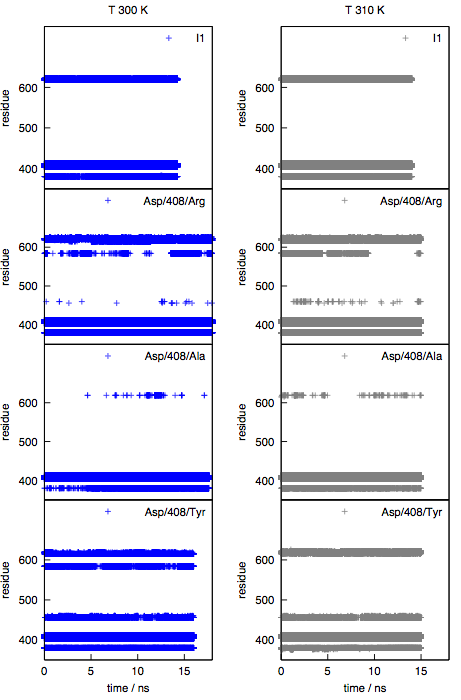


**Fig G. Map contacts for the I1 variant and the M1-3 mutants at 300K and 310K.** Analysis performed for the last 15 ns of the R0 simulation trajectory. The I1 variant showed stable contacts with residues at TM helix 7, whereas M1 almost lost the interactions, and M2 and M3 showed additional contacts involving TM helices 4 and 6 not present in the I1 variant .


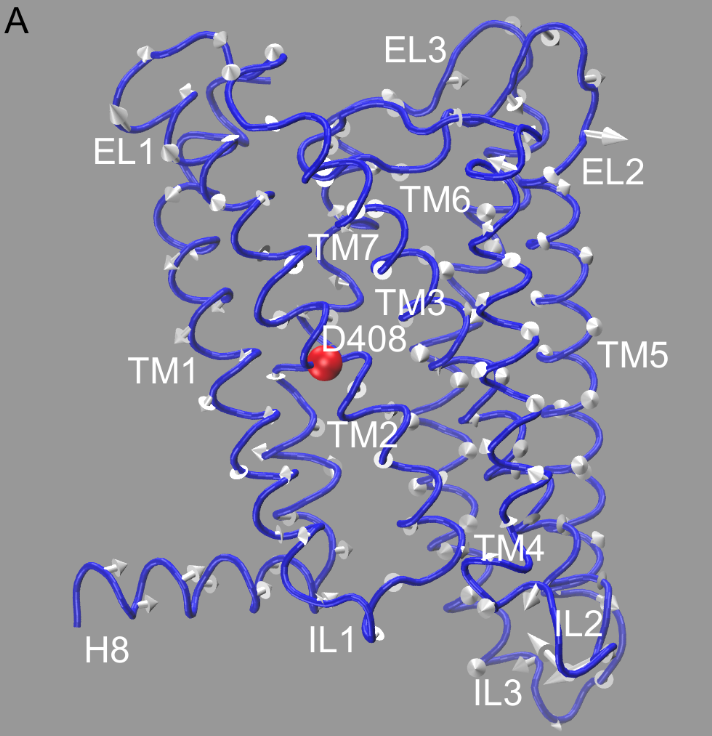

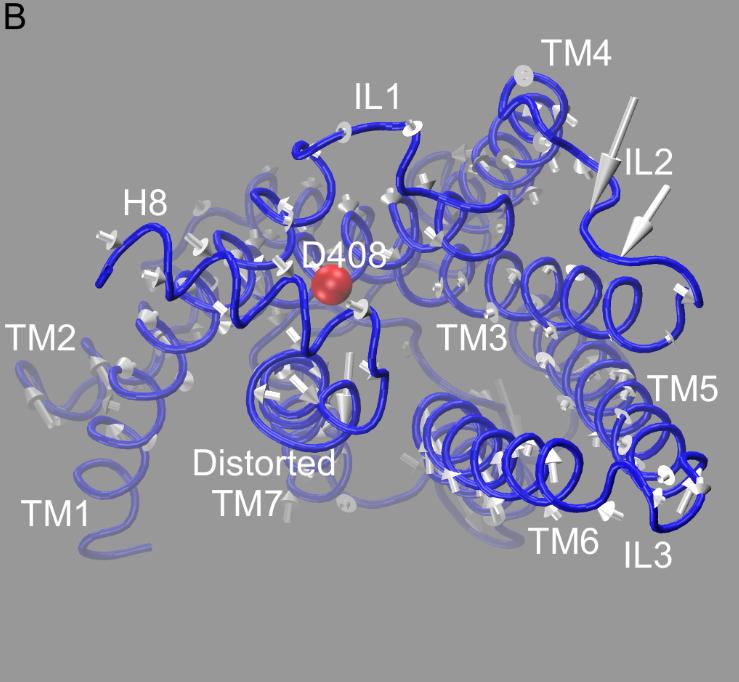


**Fig H**. **Motion of the TM domains along the PC1 for the I2 FSHR variant**. (A) Side view of the TM domains showing the initial conformation of the I2 variant (blue ribbons) along the PC1 axis. D408 (red sphere) is located in TM2. White arrows represent the motion of the Cα atoms and define both direction and distance spanned. The amplitude of the motion over the PC1 was smaller in I2 in comparison to the amplitude of the I1 FSHR variant. (B) Intracellular view of the I2 variant showing the receptor conformation at the end of the PC1 motion. The motion of H8 toward TM helix 7 caused a distortion of the last turn of the helix


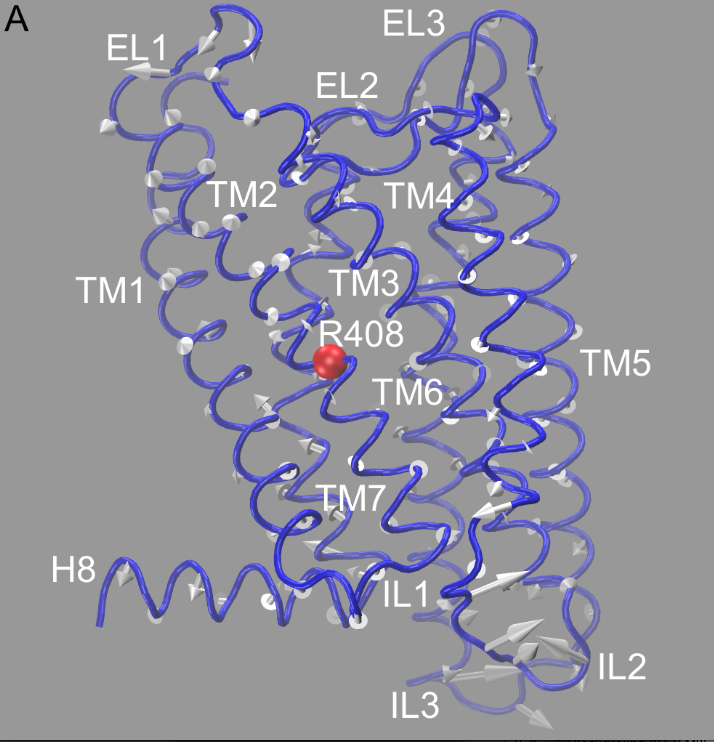

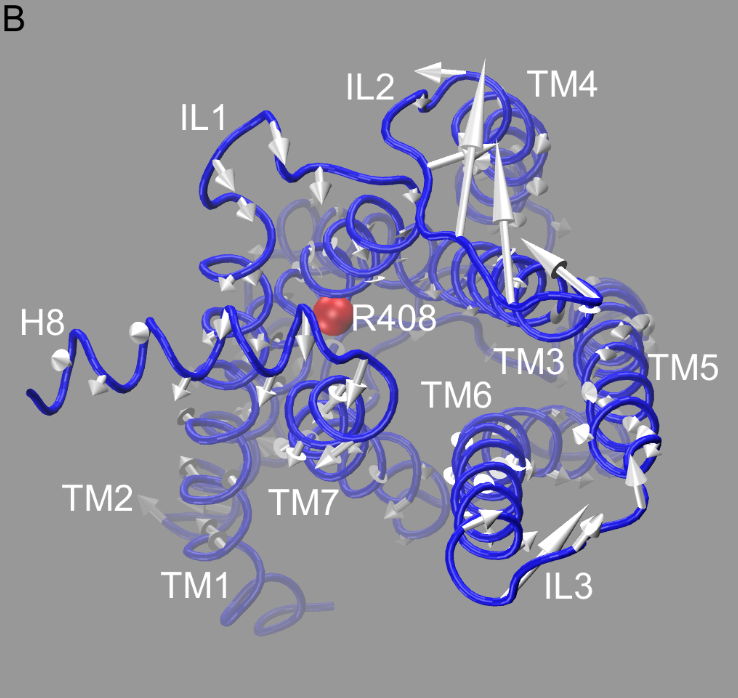


**Fig I**. **Motion of the TD domains along the PC1 for the M2 FSHR mutant**. (A) Side view of the TM domains showing the initial conformation of the M2 mutant (blue ribbons) along the PC1 axis. R408 (red sphere) is located in TM2. White arrows represent the motion of the Cα atoms and define both direction and distance spanned. (B) Intracellular view of the M2 mutant. Concerted motion of TM helix 1-2, the upper half of TM helix 3, and TM helix 7 and helix 8 was detected. The motion was in opposite direction of the TM helix 6 and the loops IL2 and IL3.


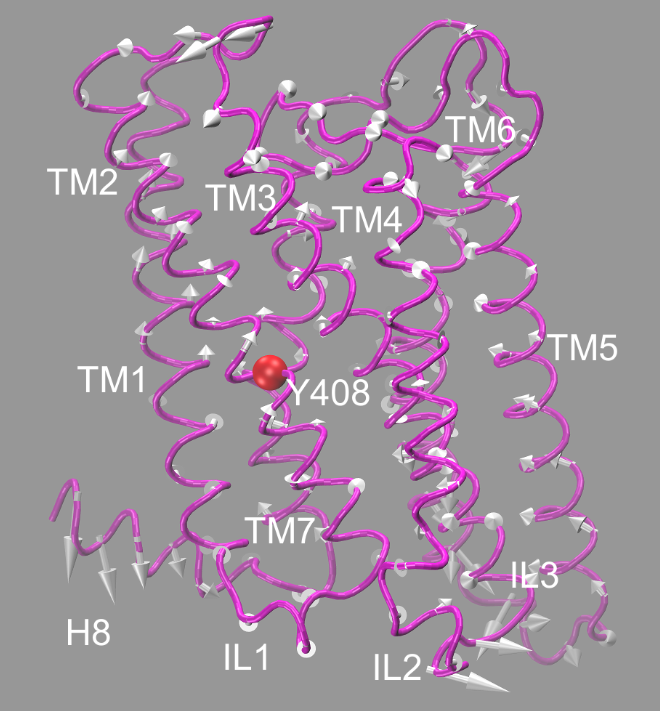


**Fig J**. **Motion of the TD domains along the PC1 for the M3 FSHR mutant**. Side view of the TM domains showing the initial conformation of the M3 mutant (magenta ribbons) along the PC1 axis. Y408 (red sphere) is located in TM2. White arrows represent the motion of the Cα atoms and define both direction and distance spanned.

| I1 | I2 | scale |
| --- | --- | --- |
| 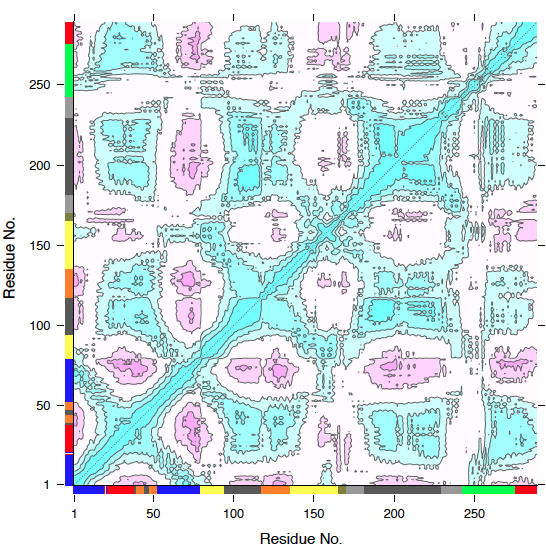 | 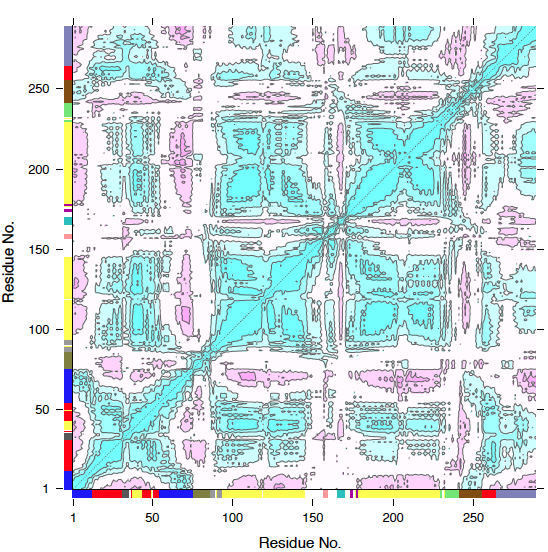 | 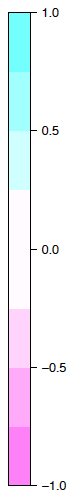 |
| M1 | M2 | M3 |
| 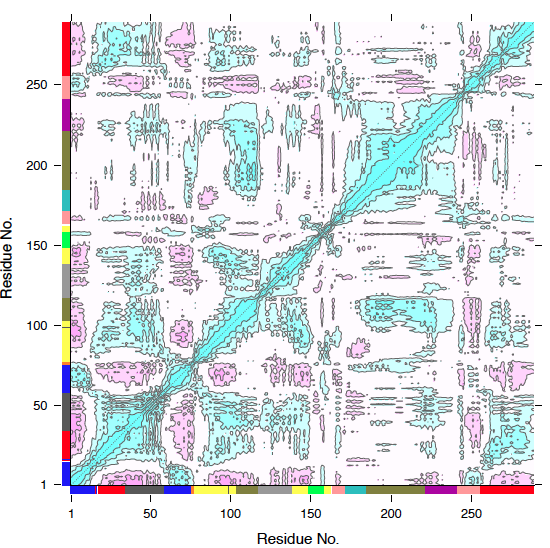 | 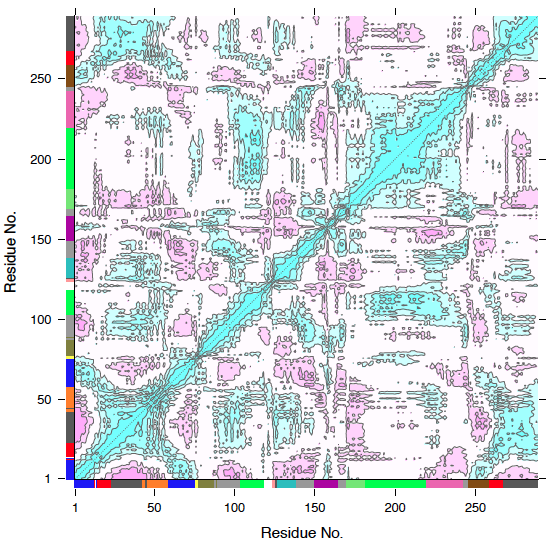 | 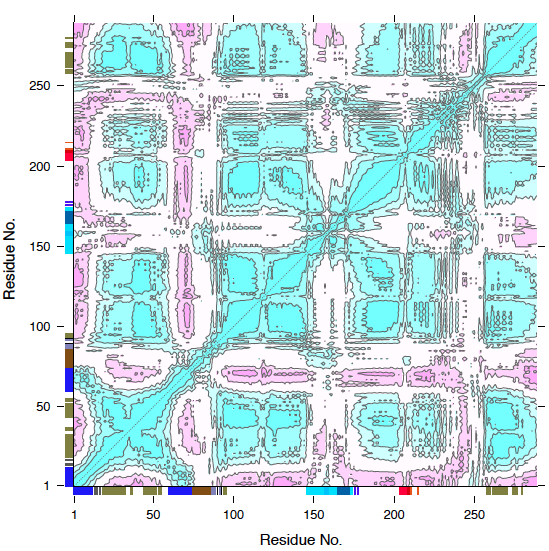 |

**Fig K. Chart for dynamical cross correlation matrices for the FSHR phenotypes.** Color scale for the correlation coefficients shown in the upper right corner for values from -1 to 1. The colors on the x- and y-axis represent the communities of the network. The network analysis was performed on these matrices to determine the network connectivity among the communities.

The elements of the dynamical cross correlation matrix (DCCM) were calculated as

where *r_i_* corresponds to the coordinates of atom *i*, and < *r_i_* > corresponds to the average position of atom *i.* Averages were calculated over time frames taken every 100 ps of the trajectories at 300 K, and 310 K. The plots corresponding to the *D_ij_*, for each phenotype, are shown below.

**Table B. Number of atoms, simulation box transversal area, and trajectory length.**

| System | Number of atoms | T=300K | |  | T=310K | |
| --- | --- | --- | --- | --- | --- | --- |
|  |  | Box Area /Å^2^ | Trajectory length / ns |  | Box Area /Å^2^ | Trajectory length / ns |
| I1  R0^a^  R1^b^  R2^c^  R3^d^ | 128982 | 9577.0 +/- 104.3 | 70.654 |  | 9612.01 +/- 86.2  9559.9 +/- 99.6  9587.0 +/- 75.2  9511.6 +/- 71.0 | 51.516  21.000  21.000  21.000 |
| I2  R0^a^  R1^b^  R2^c^ | 128979 | 9535.3 +/- 105.3 | 64.308 |  | 9667.8 +/- 98.3  9606.4 +/- 128.4  9572.2 +/- 111.2 | 52.500  22.500  22.500 |
| M1  R0^a^  R1^b^  R2^c^ | 128994 | 9550.6 +/- 79.8 | 66.663 |  | 9764.05 +/- 85.5  9673.7 +/- 108.1  9606.3 +/- 103.5 | 52.074  22.470  22.500 |
| M2  R0^a^  R1^b^  R2^c^ | 128979 | 9543.7 +/- 98.2 | 63.121 |  | 9692.4 +/- 106.0  9589.5 +/- 109.3  9652.6 +/- 91.8 | 52.500  22.500  22.500 |
| M3  R0^a^  R1^b^  R2^c^ | 128990 | 9518.2 ± 89.5 | 70.805 |  | 9684.9 ± 80.3  9696.8 +/- 116.8  9640.59 +/- 92.7 | 52.734  22.500  22.343 |

^a^ Trajectory with initial time frame at 0 ns. Calculation for the last 20 ns of trajectory.

^b^ Replicated trajectory initialized using the time frame at 30 ns of R0.

^b^ Replicated trajectory initialized using the last time frame of the R0 trajectory

^c^ Replicated trajectory initialized using the time frame at 37 ns.

**Table C. Parameters for local regression planes for the motion of Ca atoms projected over the first principal component.** Each row corresponds to a plane fitted to the movement of a given Cα projected over the principal component (PC1), for all I1, I2 and M3 phenotypes. Data for the Cα of the TM helices 1-7, and helix 8, classified as top or bottom according to its location in the upper or lower bilayer leaflet, respectively. For each plane, zero-ordinate (intercept) as well as slopes in the x- and y-axis are given. Boldface numbers indicate the parameter that differ from I1 (p<0.05).

|  |  | I1 | | |  | I2 | | |  | I3 | | |
| --- | --- | --- | --- | --- | --- | --- | --- | --- | --- | --- | --- | --- |
|  | Cα | intercept | x | y |  | intercept | x | y |  | intercept | x | y |
| TM1 | Top | 63.05 | 0.77 | 1.88 |  | **47.72** | **-0.08** | **1.33** |  | **29.94** | **-4.46** | **0.47** |
| TM1 | Bottom | -28.91 | 0.57 | 0.11 |  | **-25.87** | 0.17 | **73.32** |  | **-29.52** | **-26.78** | 0.15 |
| TM2 | Top | -12.55 | 0.02 | 0.67 |  | **-15.51** | 0.14 | **-18.95** |  | **-13.83** | **-53.40** | **0.36** |
| TM2 | Down | 24.18 | -0.35 | -0.20 |  | **4.18** | **-0.12** | **-3.57** |  | **35.10** | **39.73** | 0.28 |
| TM3 | Top | 45.79 | -0.75 | 0.82 |  | **5.70** | **-0.12** | **33.48** |  | **31.97** | **-19.29** | **0.72** |
| TM3 | Bottom | -30.57 | -0.86 | 0.65 |  | **-16.86** | **-0.16** | **55.73** |  | **-26.35** | **-3.85** | **0.66** |
| TM4 | Top | -19.47 | 0.47 | 0.50 |  | **-16.61** | 0.09 | -19.96 |  | **-20.35** | **45.84** | **0.48** |
| TM4 | Bottom | 33.28 | -0.56 | -1.19 |  | **8.07** | 0.35 | -8.97 |  | **35.49** | **41.60** | **-1.54** |
| TM5 | Top | -13.37 | -0.34 | 2.37 |  | **18.13** | 0.10 | **42.99** |  | **37.62** | **-18.15** | **-2.59** |
| TM5 | Bottom | -29.86 | -0.72 | -0.30 |  | **-19.99** | **-0.28** | **-3.46** |  | **-28.27** | **-10.49** | **0.47** |
| TM6 | Top | -31.00 | -0.54 | -0.37 |  | **-26.65** | **-0.29** | -19.49 |  | **-36.40** | **45.02** | **0.38** |
| TM6 | Bottom | 31.11 | 0.62 | -0.73 |  | **21.87** | 0.10 | **-21.08** |  | **21.27** | **46.63** | **-1.63** |
| TM7 | Top | 17.96 | 0.53 | -0.45 |  | **12.90** | 0.50 | **40.42** |  | **32.35** | **-16.75** | **1.07** |
| TM7 | Bottom | -8.44 | 0.07 | 0.18 |  | -15.29 | 0.43 | **27.32** |  | -10.05 | -26.01 | **0.04** |
| H8 | Start | -21.18 | -0.34 | -0.04 |  | **-37.51** | **-0.16** | **0.45** |  | **-19.26** | **31.42** | **0.04** |
| H8 | End | -6.89 | -0.35 | 0.49 |  | **-63.60** | **0.91** | **-12.24** |  | --- | --- | --- |


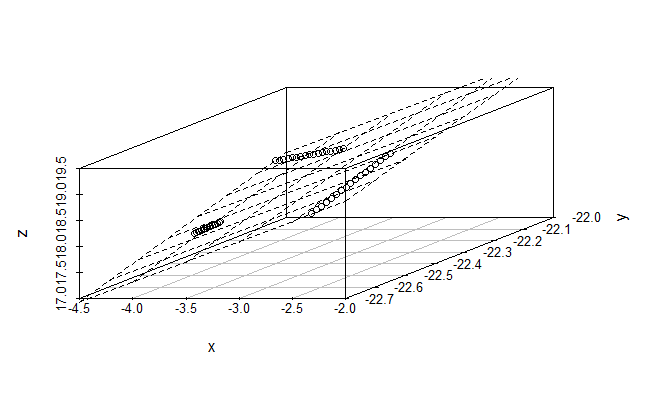


*z* = 63.05 + 0.77 *x* + 1.88 *y*

**Fig L. Parameterized plane for R0-2 of the first Cα in the S12 Table**. For each Cα in table S3, the parameters can be translated into a plane that best fit the motions projected over the PC1 axis of the PCA analysis. Parameters were compared using the technique of including categorical (dummy) variables.
